# SCR106 splicing factor modulates abiotic stress responses by maintaining RNA splicing in rice

**DOI:** 10.1093/jxb/erad433

**Published:** 2023-10-31

**Authors:** Abdulrahman Alhabsi, Haroon Butt, Gwendolyn K Kirschner, Ikram Blilou, Magdy M Mahfouz

**Affiliations:** Laboratory for Genome Engineering and Synthetic Biology, Division of Biological Sciences, 4700 King Abdullah University of Science and Technology, Thuwal 23955-6900, Saudi Arabia; Laboratory for Genome Engineering and Synthetic Biology, Division of Biological Sciences, 4700 King Abdullah University of Science and Technology, Thuwal 23955-6900, Saudi Arabia; Laboratory of Plant Cell and Developmental Biology, Division of Biological Sciences, 4700 King Abdullah University of Science and Technology, Thuwal 23955-6900, Saudi Arabia; Laboratory of Plant Cell and Developmental Biology, Division of Biological Sciences, 4700 King Abdullah University of Science and Technology, Thuwal 23955-6900, Saudi Arabia; Laboratory for Genome Engineering and Synthetic Biology, Division of Biological Sciences, 4700 King Abdullah University of Science and Technology, Thuwal 23955-6900, Saudi Arabia; Oklahoma State University, USA

**Keywords:** Abiotic stress, alternative splicing, genome engineering, pre-mRNA splicing, SR proteins

## Abstract

Plants employ sophisticated molecular machinery to fine-tune their responses to growth, developmental, and stress cues. Gene expression influences plant cellular responses through regulatory processes such as transcription and splicing. Pre-mRNA is alternatively spliced to increase the genome coding potential and further regulate expression. Serine/arginine-rich (SR) proteins, a family of pre-mRNA splicing factors, recognize splicing *cis*-elements and regulate both constitutive and alternative splicing. Several studies have reported SR protein genes in the rice genome, subdivided into six subfamilies based on their domain structures. Here, we identified a new splicing factor in rice with an RNA recognition motif (RRM) and SR-dipeptides, which is related to the SR proteins, subfamily SC. OsSCR106 regulates pre-mRNA splicing under abiotic stress conditions. It localizes to the nuclear speckles, a major site for pre-mRNA splicing in the cell. The loss-of-function *scr106* mutant is hypersensitive to salt, abscisic acid, and low-temperature stress, and harbors a developmental abnormality indicated by the shorter length of the shoot and root. The hypersensitivity to stress phenotype was rescued by complementation using OsSCR106 fused behind its endogenous promoter. Global gene expression and genome-wide splicing analysis in wild-type and *scr106* seedlings revealed that OsSCR106 regulates its targets, presumably through regulating the alternative 3'-splice site. Under salt stress conditions, we identified multiple splice isoforms regulated by OsSCR106. Collectively, our results suggest that OsSCR106 is an important splicing factor that plays a crucial role in accurate pre-mRNA splicing and regulates abiotic stress responses in plants.

## Teaser

OsSCR106, a splicing factor that is related to the SC-subfamily of SR proteins, plays an essential role in abiotic stress tolerance by controlling alternative gene splicing in rice.

## Introduction

Plants adapt to stresses with different molecular mechanisms that mainly regulate gene expression under stressful conditions (Reddy *et al.*, 2013). Pre-mRNA splicing is one of the processes that regulate gene expression under abiotic stress conditions and enhances the plasticity of the genome. RNA splicing eliminates non-coding regions (introns) in constitutive splicing, producing a single transcript (Will and Luhrmann, 2011). However, alternative splicing (AS), common in plants, produces multiple isoforms from a single gene, increasing the transcriptome and potential proteome complexity. AS generates transcripts with retained introns (RIs), skipped exons (ESs), alternative 5ʹ-splice sites (A5SSs), alternative 3ʹ-splice sites (A3SSs), or mutually exclusive exons (MXEs) (Reddy *et al.*, 2013; Laloum *et al*., 2018; Ganie and Reddy, 2021).

RNA splicing is carried out by the spliceosome, a large ribonucleoprotein complex. The U2-type spliceosome is the primary complex, with five uridine-rich small nuclear ribonucleoprotein particles (snRNPs), U1, U2, U5, and U4/U6, and many non-snRNPs (Will and Luhrmann, 2011; Shi, 2017; Gehring and Roignant, 2021; Marasco and Kornblihtt, 2022). For RNA splicing, spliceosomal components assemble to bind the respective pre-mRNA and remove introns marked by 5ʹ- and 3ʹ-splice sites, branch sites, and polypyrimidine tracts (Keren *et al*., 2010). In plants, chemical or genetic interference with spliceosome activity can inhibit the splicing, leading to cell death (AlShareef *et al.*, 2017; Ling *et al.*, 2017; Butt *et al.*, 2019a, 2021). Many factors facilitate the spliceosome assembly at the pre-mRNA, including serine/arginine-rich (SR) proteins, which recognize and bind splicing regulatory sites in pre-mRNA, regulating the splicing (Shi, 2017; Morton *et al.*, 2019; Di Zhang *et al*., 2020; Gehring and Roignant, 2021; Marasco and Kornblihtt, 2022).

The SR protein family is a conserved RNA-binding protein family among metazoans, plants, fungi, and protozoa (Plass *et al.*, 2008; Richardson *et al.*, 2011). Plant SR proteins were discovered by investigating sequence homology to mammalian SR proteins or interactions with U1 snRNP [70K, (U1-70K)] (Birney *et al*., 1993; Lazar *et al.*, 1995; Golovkin and Reddy, 1998). There are two SR proteins in fission yeast (Barbosa-Morais *et al*., 2006), 12 in humans (Manley and Krainer, 2010), 18 in Arabidopsis, and 22 in rice (Richardson *et al.*, 2011). They have also been identified in algae, maize, wheat, soybean, sorghum, grape, and other plants (Rauch *et al.*, 2014; Chen *et al.*, 2019; Di Zhang *et al*., 2020). An SR protein contains either a single or a double N-terminal RNA recognition motif (RRM) and a C-terminal arginine/serine-rich (RS) domain for protein–protein interactions (Barta *et al*., 2010; Di Zhang *et al*., 2020). The phosphorylation state of residues in RS domains can influence protein interaction, subcellular localization, and splicing activity of SR proteins (Stamm, 2008; Xiang *et al.*, 2013; Zhou and Fu, 2013). SR proteins are classified into six subfamilies, among which SR, SC, and RSZ are conserved between humans and plants, whereas RS, SCL, and RS2Z are plant specific (Barta *et al*., 2010; Reddy and Shad Ali, 2011; Morton *et al.*, 2019). Interestingly, the widespread occurrence of SR proteins in higher eukaryotes corresponds with elevated AS complexity, suggesting their essential role in regulating complex splicing events (Busch and Hertel, 2012). Moreover, SR proteins are employed in many post-transcriptional processes, including mRNA movement, stability, and translation (Jeong, 2017). SR proteins are highly dynamic and mainly localized to nuclear speckles (Caceres *et al.*, 1997), in which splicing factors are stored before recruitment to splicing events in the nucleoplasm (Misteli *et al*., 1997; Galganski *et al*., 2017). Many SR proteins shuttle back and forth between the nucleus and cytoplasm (Caceres *et al*., 1998).

Investigations on plant SR proteins have emerged more slowly than in animals, and most of the studies have linked plant AS to physiological and stress responses (Carvalho *et al*., 2013; Staiger and Brown, 2013; Laloum *et al*., 2018). The expression pattern of SR genes changed in response to development or stress conditions, indicating possible involvement in regulating these conditions (Duque, 2011; Reddy and Shad Ali, 2011; Filichkin *et al.*, 2015; Jiang *et al.*, 2017). The Arabidopsis loss-of-function mutant *sr45-1* has provided insights toward understanding the physiological role of plant SR proteins (Di Zhang *et al*., 2020). *sr45-1* exhibited developmental abnormalities (Ali *et al.*, 2007) and was sensitive to sugar, abscisic acid (ABA), and salt treatment (Carvalho *et al.*, 2010, 2016; Albaqami *et al*., 2019). *SR45* produced two isoforms; the SR45.1 isoform was involved in flower development, whereas SR45.2 was involved in root growth (Zhang and Mount, 2009; Albaqami *et al*., 2019). The loss-of-function mutants of other members of the SR family, such as *rs40*, *rs41*, and *scl30a*, showed hypersensitivity to salt and ABA stresses in *Arabidopsis thaliana* (Chen *et al*., 2013; Laloum *et al*., 2023).

In rice, abiotic stresses such as ABA, NaCl, heat, and cold induce AS of SR transcripts (Zhang *et al.*, 2013). SR proteins maintain nutrient homeostasis, such as phosphate remobilization by SR40, SCL25, and SCL57 in rice shoots (Dong *et al.*, 2018). Recently, functional investigation analysis showed that the *rs33* loss-of-function rice mutant was hypersensitive to salt and low-temperature stresses, and that *RS33* regulates pre-mRNA splicing in response to abiotic stresses (Butt *et al.*, 2019b, 2022). In contrast, no morphological changes were observed in rice after overexpressing several SR genes individually, including *SR32*, *SR33a*, *SR33*, *SCL26*, *RSZ23*, *RS2Z36*, and *RS2Z37*, although lines overexpressing *RS29* and *RS33* were lethal as they could not be recovered (Isshiki *et al*., 2006). Moreover, increased expression of *SCL30* compromised salt, drought, and low-temperature tolerance in rice (Wu *et al.*, 2022). In addition, single and multiplex mutants of SR protein genes were created using the CRISPR/Cas 9 [clustered regularly interspaced palindromic repeats (CRISPR)/CRISPR-associated protein 9] system and may be utilized to understand the role of SR proteins in plant development and stress response (Butt *et al.*, 2019b). Overall, the above studies indicate that SR proteins regulate the pre-mRNA splicing under abiotic stress conditions; thus, accurate and efficient regulation of these splicing factors may increase the adaptation to abiotic stress. However, most of these proteins are not well characterized, and the role of a single SR protein and the splicing events they regulate is also not yet clear.

In the present study, we characterized a novel splicing factor termed OsSCR106 in rice. The phylogenetic analysis showed that this protein is rich in SR dipeptides and contains an RRM domain at its N-terminus. Expression analysis showed that *OsSCR106* is expressed in seed endosperm, leaves, and vascular tissues. The *OsSCR106:EGFP* fusion under the *OsUbiquitin* promoter localized to the nuclear speckles. We investigated the function of *SCR106* via targeted mutagenesis using the CRISPR/Cas9 system. The *scr106* mutant, like mutants of other SR proteins, is hypersensitive to abiotic stress conditions such as salt and low temperature. These hypersensitive phenotypes were rescued via the expression of *OsSCR106* cDNA. RNA-seq analysis identified a subset of genes involved in salt stress responses, which are regulated by *OsSCR106*. Our results identified OsSCR106 as a splicing factor and prove its critical role in regulating AS and determining stress responses.

## Materials and methods

### Plant materials

*Oryza sativa* L. ssp. *japonica* cv. Nipponbare was used for all experiments. All plants were grown at 28 °C in the KAUST greenhouse under natural day length. Plants were watered twice a week and supplemented with Hoagland solution biweekly.

### Vector construction

For targeted mutagenesis, the expression of Cas9 was driven by *OsUbiquitin*, and the single guide RNA (sgRNA) was expressed under the *OsU3* promoter. The sgRNAs were designed to target the first exon of *OsSCR106* (*LOC_Os01g01150*; *Os01g0101600*) in rice. The *pRGEB32* plasmid (Xie *et al*., 2015; Butt *et al.*, 2019b) was digested with *Bsa*I; sgRNAs were synthesized as oligonucleotides with *Bsa*I overhangs, GGCA in the forward oligonucleotides and AAAC in the reverse. The oligonucleotides were annealed and ligated in the *Bsa*I-digested vector.

To generate the overexpression construct tagged with green fluorescent protein (GFP), *pUBI::OsSCR106-EGFP*, we amplified the full-length CDS of *OsSCR106* and cloned it into the binary vector pENTR™/D-TOPO™ (Thermo Fisher Scientific) according to the manufacturer’s instructions. The GFP sequence is amplified using primers EcoRI_GFP_F and KpnI_GFP_R to clone in-frame with *OsSCR106.* Using gateway cloning, an LR reaction was performed to transfer OsSCR106-EGFP into the destination vector pRGEB32.

To generate the promoter analysis construct, *pOsSCR106::EGFP-GUS*, we amplified ~2.5 kb upstream and 45 bp downstream of the start codon (ATG) of *OsSCR106*. The PCR products were then cloned into the binary vector pENTR™/D-TOPO™ according to the manufacturer’s instructions. Then we performed an LR reaction to transfer *pOsSCR106* in-frame with EGFP–β-glucuronidase (GUS) into the destination vector pKGWSF7 (Karimi *et al*., 2002).

To generate the complementation construct, *pOsSCR106::OsSCR106-EGFP*, we amplified and cloned *pOsSCR106* by restriction digestion into pRGEB32 by replacing the *OsUbiquitin* promoter sequence. *OsSCR106-EGFP* was amplified from the vector *pUBI::OsSCR106-EGFP* and cloned downstream of *pOsSCR106* in the pRGEB32 destination vector.

### Rice transformation

*Agrobacterium*-mediated rice transformation was performed as described previously (Hiei and Komari, 2008; Butt *et al.*, 2019b). The *Agrobacterium tumefaciens* strain EHA105 was used for all transformations. For the pRGEB32 vector, 50 mg l^–1^ hygromycin was used for selection and regeneration. For the pKGWSF7 vector, 150 mg l^–1^ G418 was used for selection and 100 mg l^–1^ G418 was used for regeneration.

### Genotyping of the *OsSCR106* mutant plants

DNA was extracted from rice leaves frozen in liquid nitrogen during collection. PCR was performed using the gene-specific primers to amplify the gRNA-targeted region. PCR product size was confirmed by gel electrophoresis and gel purified, then cloned into pJET1.2 blunt vector using the CloneJET PCR Cloning Kit (K1231). The clones were then Sanger sequenced to analyze the mutations.

### Phenotypic analysis of mutants and complementation lines under abiotic stress conditions

We used homozygous knockout (k/o), Cas9 free, and T_2_ or T_3_ generations for all phenotypic analysis. Freshly harvested seeds of each genotype were used for stress tolerance assays. The sterilized seeds were transferred to half-strength Murashige and Skoog (1/2 MS) solid medium supplemented or not with 100 mM NaCl, 125 mM NaCl, 2 µM ABA, 5 µM ABA, 100 mM mannitol, and 200 mM mannitol. The plates were sealed and incubated in a plant growth chamber maintained at 28/26 °C, 16/8 h day/night photoperiod. The seedlings were allowed to grow vertically for 7 d. The control conditions are without NaCl, ABA, and mannitol.

To test the low-temperature tolerance of these genotypes, Petri plates were transferred to different growth chambers maintained at the indicated temperatures for a specified time. The seeds initially subjected to the low-temperature treatment of 16 °C and 4 °C were transferred to 28/26 °C after 14 d, for 5 d. The seeds were allowed to grow for 12 d at 28/26 °C, 22/20 °C, or 18/15 °C. The control is normal growth conditions at 28/26 °C.

### Histochemical analysis of *OsSCR106* gene expression

Transgenic seedlings or plant organs carrying GUS reporter constructs were harvested, dipped in 3 ml of GUS staining buffer in a 6-well culture plate, and incubated at 37 °C. If necessary, a vacuum was applied for 10 min at room temperature to facilitate buffer uptake. When staining was complete, the samples were rinsed with dH_2_O and de-stained by shaking overnight at room temperature in 70% ethanol (EtOH). The plant material was rehydrated by successive 15 min incubation in 40, 20, and 10% (v/v) EtOH followed by an overnight incubation in 5% (v/v) EtOH/25% (v/v) glycerol.

### Subcellular localization analysis

Roots of *pUBI::OsSCR106-EGFP* and *pOsSCR106::OsSCR106-EGFP* were fixed in 4% paraformaldehyde in phosphate-buffered saline (PBS) for 2 h, washed with dH_2_O and incubated in ClearSee solution (Kurihara *et al.*, 2015) for ~2 weeks, and then imaged with the Zeiss LSM710 inverted microscope.

### RNA isolation and sequencing

Total RNA was extracted from 7-day-old rice seedlings grown in 1/2 MS media with or without 125 mM NaCl using the Direct-zol RNA MiniPrep Plus kit (Zymo Research). We followed the manufacturer’s recommendations. RNA was quantified using a Nanodrop, and RNA quality was examined using a 2100 Bioanalyzer (Agilent Technologies). High-quality RNA samples with RNA integrity number (RIN) ≥7.0 were selected for library construction. The RNA-seq libraries were constructed using the TruSeq mRNA stranded kit following the standard protocol and sequenced on the Nova-seq platform to generate high-quality paired-end reads.

### Analysis of RNA-seq data and gene functional classification

RNA-seq data analysis was performed by Sequentia Biotech®. The dataset included RNA-seq data from 12 samples belonging to two genotypes (*scr106* and the wild type), two experimental conditions (salt stress, N; and control, C), and three biological replicates.

The quality of the reads was assessed with the software FASTQC, then a trimming step was performed in order to remove adapters and low-quality bases from the reads. The following parameters were used: minimum length was set to 35 bp and the quality score to 25. The software TRIMMOMATIC was used for this purpose. On average, 43.5 million filtered reads were obtained per sample. The high-quality reads were aligned against the *O. sativa* cv. Nipponbare genome (IRGSP 1.0) with STAR aligner (version 2.7.10a). On average, 88.95% of the reads could be mapped uniquely on the genome. FeatureCounts (version 2.0.0) was used to calculate gene expression values as raw fragment counts. In addition, a normalization was applied to the raw fragment counts by using the Trimmed Mean of M values (TMM) and the Fragments Per Kilobase Million (FPKM) normalization. All the statistical analyses were performed with R with the packages HTSFilter and edgeR.

The edgeR package determines differential expression using empirical Bayes estimation and exact tests based on a negative binomial model. The genes showing a false discovery rate (FDR) ≤0.05 were considered to be statistically significant. For the significantly differentially expressed genes (DEGs), a Gene Ontology enrichment analysis (GOEA) was performed to identify the most enriched Gene Ontology (GO) categories across the down- and up-regulated genes.

In addition to the differential expression analysis of the genes, a differential splicing analysis was performed using the software RMATS (version 4.1.2). Only the differential events supported by an FDR ≤0.05 and an absolute IncLevelDifference >0.1 were considered.

### RT–PCR and RT–qPCR

For reverse–transcription PCR (RT–PCR), DNA digestion of total RNA samples was performed using an RNase-Free DNase Set (Invitrogen cat. no. 18 068-015) following the manufacturer’s protocol. The total RNA was reverse transcribed using a SuperScript First-Strand Synthesis System (Invitrogen) to generate cDNA. PCR conditions were: initial denaturation at 95 °C for 2 min, then 40 cycles of 95 °C for 30 s, 55 °C for 30 s, and 72 °C for 60 s, then final elongation at 72 °C for 5 min.

The quantitative RT–PCR (RT–qPCR) was performed using 50 ng of RNA in a 10 µl final reaction volume. The iTaq™ Universal SYBR® Green One-Step Kit (Bio-Rad, cat. no. 172-5150) was used, with the following manufacturer’s protocol conditions: reverse transcription 50 °C for 10 min, followed by 40 cycles of 95 °C for 15 s, 60 °C for 1 min, melt-curve analysis 65–95 °C, 0.5 °C increment. The gene expression data were normalized using the 2^–ΔΔ*C*T^ method with three biological replicates. The expression values were normalized with respect to the values of *OsActin* (*LOC_Os03g50885*). All expression analysis was performed on three biological replicates. Primers used for RT–PCR and RT–qPCR are listed in Supplementary Table S1.

## Results

### OsSCR106 is a homolog of human SRSF11 and contains plant serine/arginine protein characteristics

**Fig. 1.**
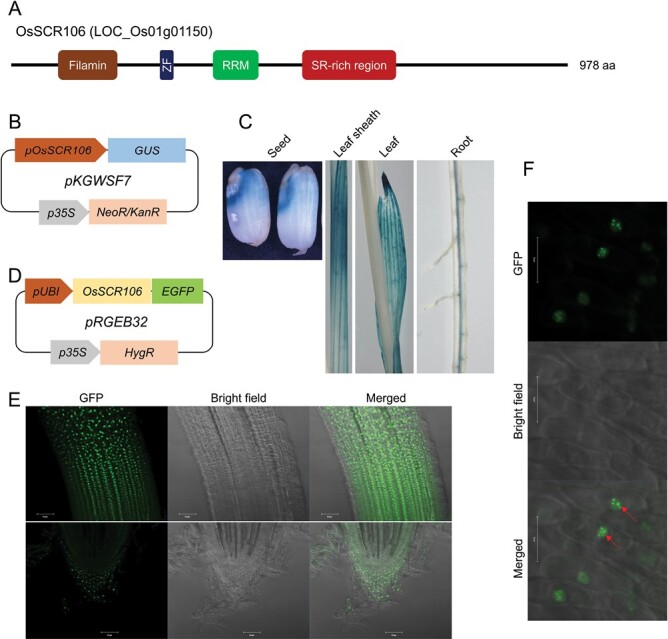
Expression analysis and subcellular localization of OsSCR106 in rice. (A) Domain organization of the OsSCR106 (SC-Related, LOC_Os01g01150) protein, generated using the publicly available online tool Prosite (https://prosite.expasy.org/). (B) Schematic diagram of the pKGWSF7 construct for expressing *pOsSCR106::GUS*. The expression is driven by the endogenous promoter of *OsSCR106*. (C) GUS staining analysis of transgenic plants carrying *pOsSCR106::GUS*. (D) Schematic diagram of the pRGEB32 construct for expressing *pUBI::OsSCR106:EGFP*. The expression is driven by the *OsUbiquitin* promoter (*pUBI*). (E) *pUBI::OsSCR106:EGFP* expression in the transition zone and the root tip of roots of 7-day-old seedlings. (F) Magnification of *OsSCR106:EGFP* expressed under pUBI in rice roots; red arrows indicate nuclear speckles. Bar, 50 µM or 20 µM in white.

In rice, only 22 SR proteins have been identified (Barta *et al*., 2010; Richardson *et al.*, 2011; Morton *et al.*, 2019). To further identify proteins that contain RS dipeptides, we used the Basic Local Alignment Search Tool (BLAST) to compare the amino acid sequence of human splicing factor SRSF11 against the Arabidopsis proteome and obtained an ortholog, At3g23900, with 32% identity. At3g23900 has been recently characterized as an immunoregulatory RNA-binding (IRR) protein with three domains, namely a zinc finger, an RRM, and an RS dipeptide (Dressano *et al.*, 2020). IRR was involved in the negative regulation of immune response in maize and Arabidopsis (Dressano *et al.*, 2020). To investigate orthologs in rice, we then used BLAST to compare the amino acid sequence of At3g23900 against the rice proteome and identified a putative, uncharacterized ortholog, LOC_Os01g01150, with almost 60% identity. We aligned the amino acid sequences of human SRSF11, Arabidopsis At3g23900, and rice LOC_Os01g01150 to investigate conserved amino acid regions. The results suggest a conserved N-terminal region with an RRM and a C-terminal region with many SR dipeptides (Supplementary Fig. S1). We generated a phylogenetic tree to investigate the evolutionary relationship among the 22 rice SR genes, including LOC_Os01g01150. The results indicated that LOC_Os01g01150 is closely related to SC-subfamily members (Supplementary Fig. S2). Then we aligned the protein sequence of LOC_Os01g01150 with rice SC-subfamily proteins and found that this new ortholog has an RRM and an RS domain like all SC-subfamily members (Supplementary Fig. S3). The RS domain in LOC_Os01g01150 contains at least 350 amino acids with almost 25% SR or RS dipeptides. In addition, the LOC_Os01g01150 protein has an N-terminal filamin and zinc finger domains which might have some additional functions (Fig. 1A). Based on the similarity to the domain structure of SR-subfamily SC and with a molecular weight of 106 kDa, we propose the name *OsSCR106* (*SC-Related106*) for the locus *LOC_Os01g01150*.

### *OsSCR106* is expressed in different rice tissues, and the protein localizes to nuclear speckles

To study the expression pattern of *OsSCR106* in different rice organs, we fused the *GUS* reporter under the 2.5 kb promoter sequence of *OsSCR106*, and stably transformed rice calli using agrobacteria (Fig. 1B). The histochemical analysis showed GUS activity in the seed endosperm, the leaf sheath, and leaves (Fig. 1C). In the root, GUS activity was detected in the vascular cylinders of both primary and lateral roots.

The major splicing activity is performed in the nuclear speckles of the cell (Shepard and Hertel, 2009). To analyze the localization of OsSCR106 *in planta*, we fused the 2.934 kb CDS of *OsSCR106* to EGFP and expressed it under the rice *OsUbiquitin* promoter (*pUBI*), stably in rice (Fig. 1D). OsSCR106:EGFP localized to nuclear speckles in rice roots (Fig. 1E, F). Broad expression of the *OsSCR106* promoter and OsSCR106 localization to nuclear speckles indicates a possible role during the pre-mRNA splicing regulation in rice.

### Targeted mutagenesis of the rice *OsSCR106* locus

**Fig. 2.**
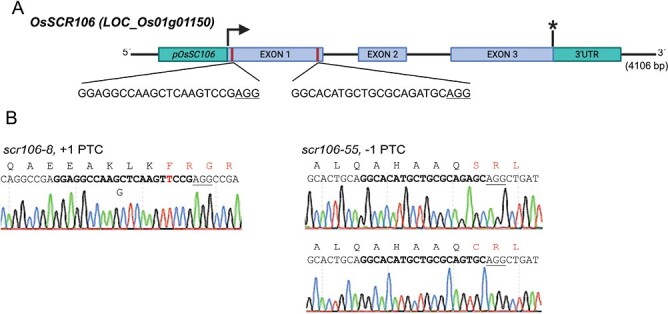
Targeted mutagenesis of the rice *SCR106* locus. (A) Schematic of the *OsSCR106* gene structure and the target site. *OsSCR106* contains three exons. The translation initiation codon (ATG) (arrow) and termination codon (TGA) (asterisk) are shown. The target site nucleotides are shown in uppercase letters, and the protospacer adjacent motif (PAM) site is underlined. (B) Nucleotide sequences and translation at target site 1 and 2 in T_0_ mutant rice plants. The recovered mutated allele sequences are shown. The PAM site is underlined. The red uppercase letters indicates the inserted nucleotides, and/or changed amino acid. ‘–/+’ indicate the deletion or insertion of the indicated number of nucleotides. The chromatogram represent data obtained by Sanger sequencing.

To further investigate the role of *OsSCR106* in rice, we targeted the *OsSCR106* locus in rice using the CRISPR/Cas9 system. The *OsSCR106* locus has three exons, and we designed two different sgRNAs to target the first exon (Fig. 2A). These sgRNAs were cloned under the *U3* promoter, and Cas9 expression was driven by the *OsUbiquitin* promoter. We delivered the plasmid into rice callus using the *Agrobacterium*-mediated transformation method. We recovered the transgenic lines and analyzed the targeted regions via Sanger sequencing. We identified several mutants in the T_0_ lines and continued with homologous k/os that contained a premature stop codon (PTC) downstream of the target site (Fig. 2B). For k/o line *scr106-8*, the insertion of 1 bp (T) at target site 1 (TS1) changes the downstream protein sequence and causes a PTC after 143 amino acids. Similarly, the k/o *scr106-55* with a deletion of 1 bp (A or T) at target site 2 (TS2) changes the downstream protein sequence and causes a PTC after 25 amino acids (Fig. 2B). We continued with k/o line *scr106-8* (*scr106*) for subsequent experiments.

### The *scr106* mutant is hypersensitive to abiotic stress conditions

**Fig. 3.**
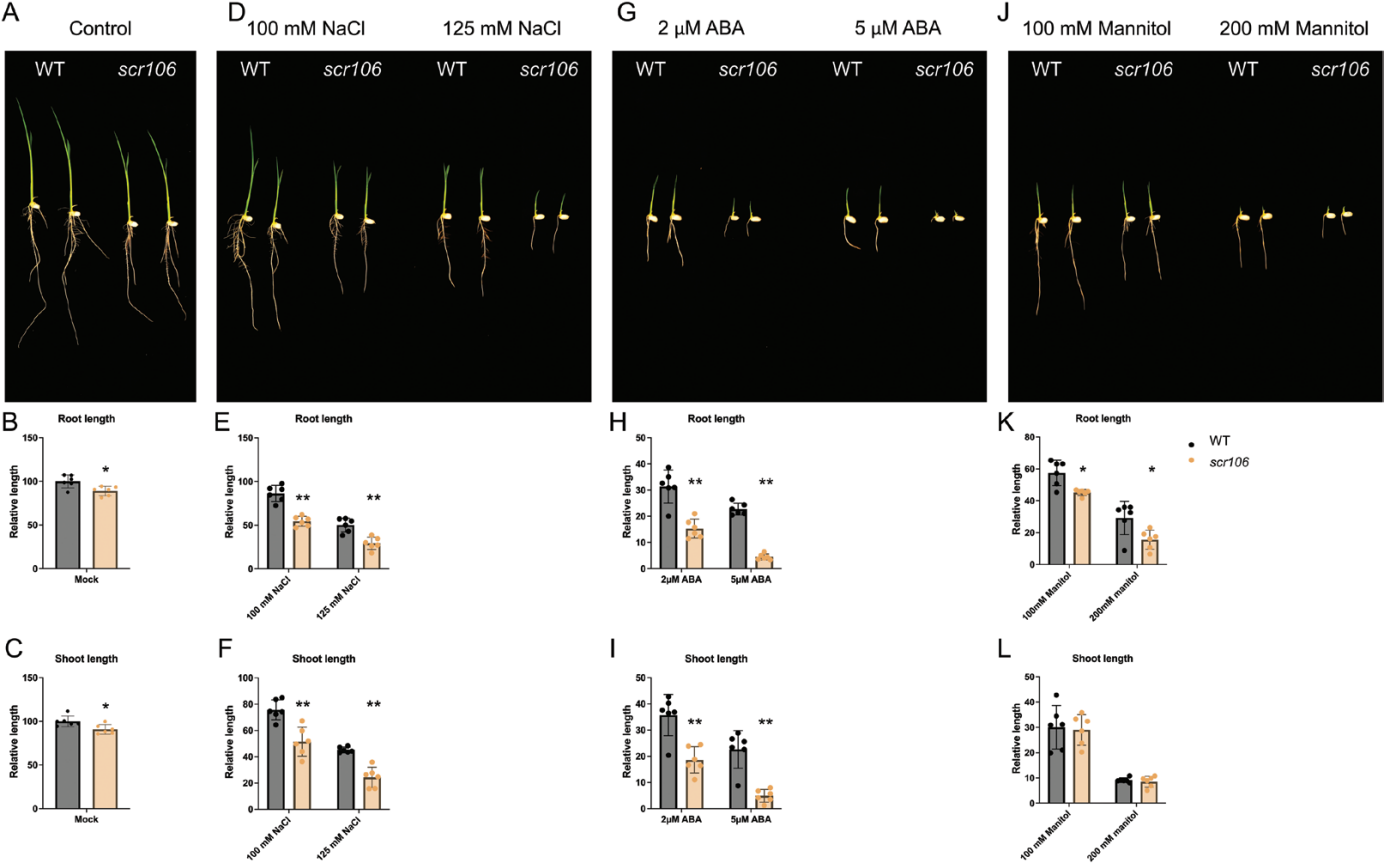
The *scr106* mutant is hypersensitive to salt, ABA, and mannitol. (A, D, G, J) Root and shoot phenotype of the wild type (WT) and *scr106* mutant grown on 1/2 MS media for 7 d, under control conditions (A), salt treatment (D), ABA treatment (G), or mannitol treatment (J). (B, C, E, F, H, I, K, L) Root and shoot length of the WT and *scr106* mutant under control and stress treatment; organ length was calculated relative to root or shoot length of the WT plants grown on 1/2 MS media. The Student’s *t*-test analysis indicated a significant difference compared with the WT (*P<0.05, **P<0.01). Values are the means ±SD of at least six biological replicates (represented as dots).

Plant SR proteins are critical modulators in abiotic stress responses, and most of the SR pre-mRNAs are alternatively spliced under abiotic stress conditions (Zhang *et al.*, 2013). The SR mutants were shown to be hypersensitive to abiotic stresses such as salt, ABA, and cold (Carvalho *et al*., 2010; Albaqami *et al*., 2019; Butt *et al*., 2022). To investigate the biological function of *OsSCR106* in rice under abiotic stress conditions, we germinated wild-type and *scr106* mutant seeds on a growth medium supplemented with different stress treatments (Fig. 3). Under control conditions, the *scr106* mutant exhibited slightly shorter root and shoot lengths than wild-type seedlings 7 d after germination. (Fig. 3A–C). This might indicate that OsSCR106 regulates the growth and development of rice plants.

We next tested the salt stress response of our mutant as compared with the wild type. For this, we used two different concentrations of NaCl, 100 mM and 125 mM (Fig. 3D–F). Root and shoot length of the mutants was significantly reduced compared with the wild-type seedlings with 100 mM NaCl treatment, and even further with 125 mM NaCl treatment, indicating that *oscr106* is hypersensitive to salt stress (Fig. 3D–F). We further tested the growth response of the wild type and *scr106* mutant under ABA stress treatment (Fig. 3G–I). Under 2 μM ABA treatment, the root and shoot lengths of *scr106* mutant seedlings were significantly shorter as compared with wild-type seedlings, and growth of *scr106* seedlings had almost ceased at 5 μM ABA (Fig. 3G–I). For osmotic stress treatments, we used mannitol at two different concentrations. Mannitol has little impact on shoot length in the *scr106* mutant as compared with the wild type (Fig. 3J–L). However, the root length was affected at 100 mM mannitol and severely reduced at 200 mM mannitol as compared with the wild type.

**Fig. 4.**
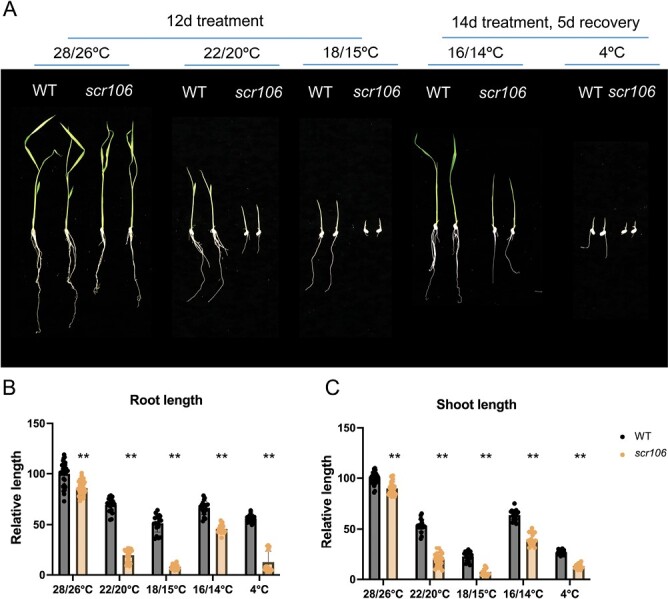
The mutation of *SCR106* leads to hypersensitive phenotypes to low-temperature treatments. (A–C). The seeds of the wild type (WT) and *scr106* mutant were germinated on 1/2 MS media and grown at different low temperatures. Seedlings were grown at (day/night) 28/26 °C, 22/20 °C, or 18/15 °C for 12 d. At 16/14 °C, or 4 °C, seeds were initially treated at these temperatures for 14 d and then recovered for 5 d. The germination and growth of the *scr106* mutant are significantly delayed compared with the WT. The relative growth length was calculated based on a root or shoot length of 100% for plants of each genotype grown on 1/2 MS media at the indicated temperatures. For 28/26 °C, the control conditions, the relative growth length is calculated based on a root or shoot length of 100% for WT plants. The Student’s *t*-test analysis indicated a significant difference compared with the WT (*P<0.05, **P<0.01). Values are the means ±SD of at least six biological replicates (represented as dots).

It has been recently shown that AS impacts the cold responses in plants, and mutants of some splicing factors are hypersensitive to low-temperature treatment (Calixto *et al.*, 2018; Lu *et al.*, 2020; Butt *et al*., 2022). To test if OsSCR106 regulates growth under low temperatures in rice, we germinated the wild type and the *scr106* mutant under various low-temperature treatments, including 22 °C and 18 °C continuously for 12 d. We observed that the continuous low temperatures of 22 °C and 18 °C significantly affected the root and shoot growth in *scr106* compared with wild-type seedlings. The growth at 18 °C is severely inhibited in *scr106* compared with wild-type seedlings (Fig. 4A–C). To test the recovery after low-temperature treatment, we used 16 °C and 4 °C for 14 d and recovery for 5 d. With the decrease in the temperature treatment, the severity of the growth inhibition increased in *scr106* as compared with wild-type seedlings (Fig. 4A–C). The treatment at 4 °C for 14 d has the highest impact on the recovery of the seedlings. The root and shoot lengths of *scr106* are severely affected compared with the wild type at 4 °C (Fig. 4A–C).

Taken together, these results suggest that *OsSCR106* is essential for abiotic stress regulation in rice.

### Expression of *OsSCR106* cDNA in the *scr106* mutant complements its function

**Fig. 5.**
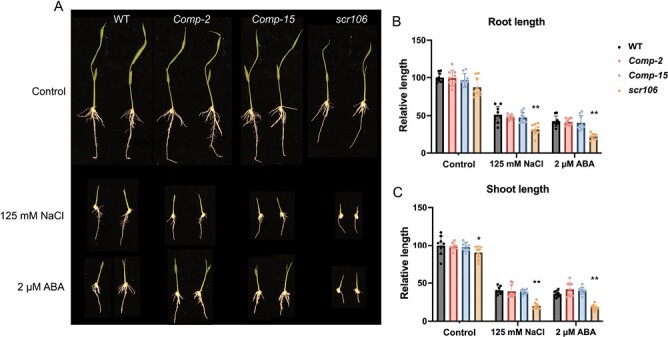
The hypersensitive phenotype of *scr106* under NaCl and ABA treatment is restored by the expression of *OsSCR106*. (A–C) The wild type (WT), the *scr106* mutant complemented with *pOsSCR106::OsSCR106-EGFP* (Comp), and the *scr106* mutant grown for 7 d under control or stress conditions. Shoot and root length of the WT, the complemented *scr106* mutant and the *scr106* mutant under control or stress conditions; length was calculated relative to root or shoot length of the WT plants grown on 1/2 MS media. The Student’s *t*-test analysis indicated a significant difference compared with the WT (*P<0.05, **P<0.01). Values are means ±SD of at least six biological replicates (represented as dots).

**Fig. 6.**
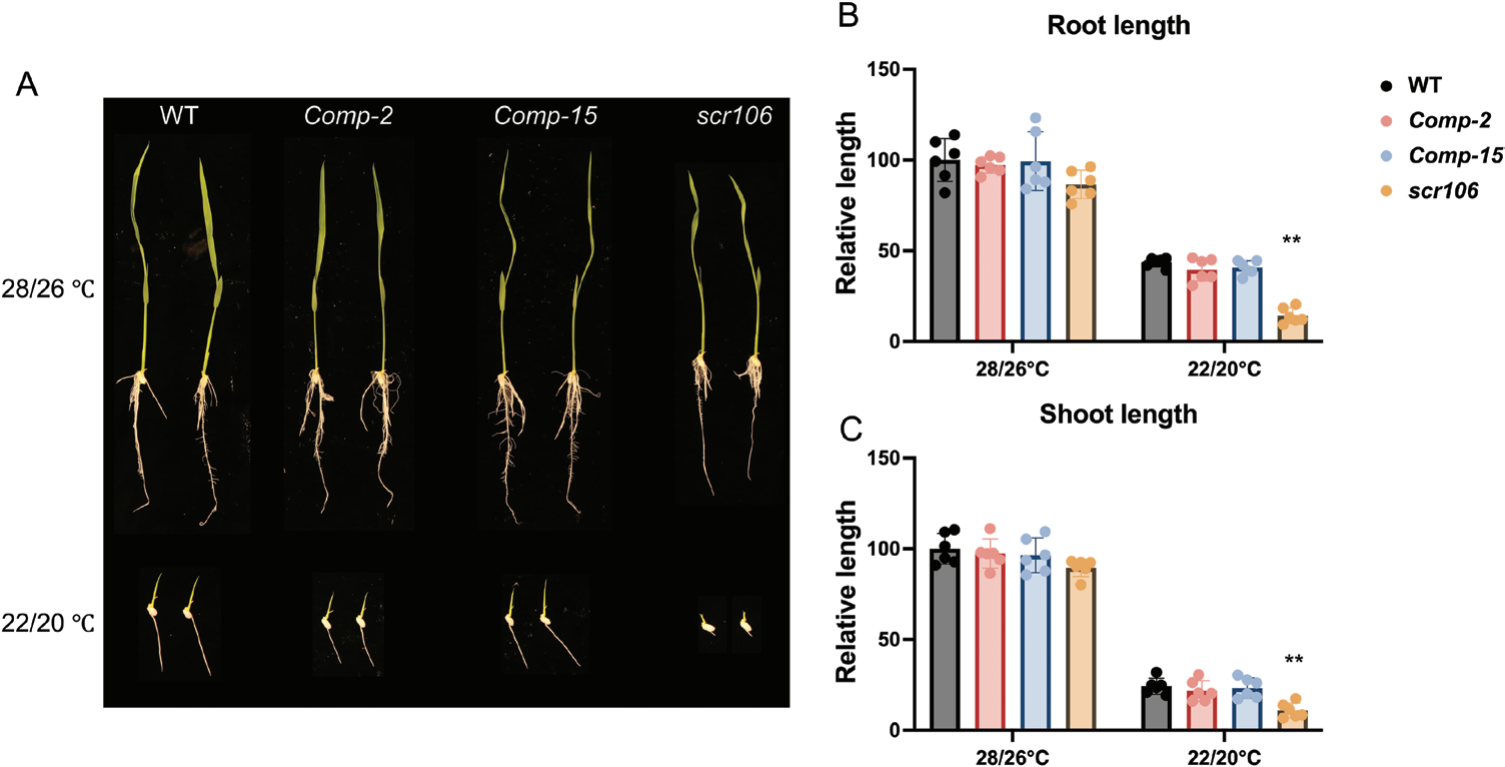
The hypersensitive phenotype of *scr106* under low temperature is rescued by the expression of *OsSCR106*. (A–C) The transformation of the *OsSCR106* cDNA rescued the hypersensitive phenotype under low temperature treatment. The relative growth length is calculated based on a root or shoot length of 100% for wild-type (WT) plants. The Student’s *t*-test analysis indicated a significant difference compared with the WT (*P<0.05, **P<0.01). Values are the means ±SD of at least six biological replicates (represented as dots).

To confirm that the loss-of-function mutation is responsible for the developmental abnormalities, and hypersensitivity to stress phenotype, we reintroduced the full-length isoform of *OsSCR106* as cDNA driven by the endogenous promoter in the mutant background. We recovered the transgenic plants and performed analysis using two independent complementation lines in the progeny. Under control conditions, the shoot and root of complementation lines showed full restoration of the wild-type phenotype (Fig. 5A–C). Furthermore, the root and shoot length of the complementation lines were fully restored to wild-type levels under salt and ABA treatment, as well as under low temperature (Fig. 6A–C). Overall, these results confirm that the hypersensitive reactions to abiotic stress treatments in the *scr106* mutant are caused by the mutation in *OsSCR106*.

### OsSCR106 regulates gene expression and controls the RNA splicing of a large number of genes in rice

**Fig. 7.**
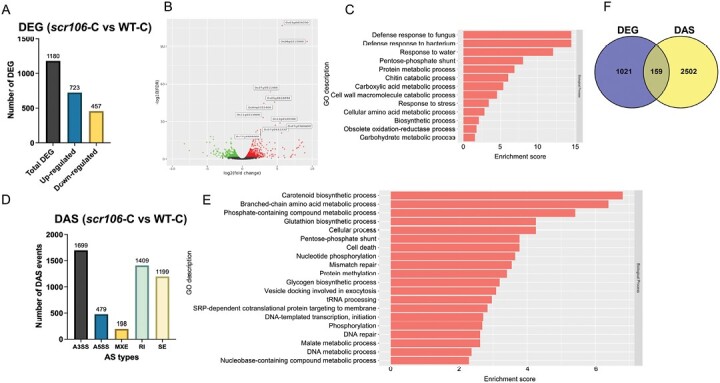
Loss of *SCR106* affects the global gene expression and alternative gene splicing in rice. (A) The number of up- and down-regulated differentially expressed genes (DEGs) in *scr106* compared with the wild type (WT). (B) The volcano plot represents the DEGs. Black dots represent the genes that are not significantly differentially expressed, while red and green dots are the genes that are significantly up- and down-regulated, respectively. The gene IDs for the top 10 highly DEGs are shown. (C) GO enrichment analysis of the DEGs regulated by *OsSCR106*. (D) Bar plot showing the differentially alternative splicing (DAS) events between *scr106* and the WT. The numbers of each type of event are given. The majority of these events are alternative 3'-splice site (A3SS), and intron retention (IR). SE, exon skipping; A5SS, alternative 5'-splice site; MXE, mutually exclusive exons. (E) GO enrichment analysis of the DAS genes regulated by *OsSCR106*. (F) Overlap of DEG with DAS events for *OsSCR106* and the WT.

To understand the role of OsSCR106 in genome-wide pre-mRNA splicing, we performed RNA-seq analysis of 7-day-old seedlings of the *scr106* mutant and wild type. Comparing the wild-type transcriptome with the *scr106* mutant identified 1180 DEGs between the wild type and *scr106*. Among these, 723 genes were up-regulated and 457 genes were down-regulated (Fig. 7A; Supplementary Dataset S1). The top 10 DEGs (based on the highest log-fold expression change) were up-regulated genes and included: *Os06g0215900*, annotated as Similar to Oxo-phytodienoic acid reductase; *Os11g0113500* annotated as retrotransposon gene 1; *Os04g0352400* is FK506-binding protein, Peptidyl-prolyl *cis*/*trans* isomerase, Chilling tolerance; *Os11g0213000* is Similar to Protein kinase domain containing protein, expressed; and *Os07g0432333* is Similar to Thionin-like peptide (Fig. 7B). The GOEA showed that the global DEGs between the wild type and the *scr106* mutant under control conditions were enriched for the biological processes related to defense response to fungus, defense response to bacterium, response to water, pentose-phosphate shunt, and protein metabolic process (Fig. 7C).

To further examine the global defects in pre-mRNA splicing regulated by OsSCR106, we compared the *scr106* and wild type, and identified differentially alternatively spliced (DAS) events. All types of events, such as A3SS, A5SS, MXE, RI, and SE, were detected. Among these, the A3SSs and the RIs were the highest DAS events between the wild type and *scr106*, with 1699 A3ʹSS and 1409 RI events, respectively (Fig. 7D; Supplementary Dataset S2). The least number of events were detected for MXEs, with 198 events. The GO enrichment analysis showed that the global DAS events between the wild type and the *scr106* mutant under control conditions were enriched in carotenoid biosynthetic process, branched-chain amino acid metabolic process, phosphate-containing compound metabolic process, glutathione biosynthetic process, and pentose-phosphate shunt (Fig. 7E). We then compared the DAS genes with DEGs and found that most of the DEGs were unique, and very few of them were alternatively spliced, uncoupling the transcription from AS regulation (Fig. 7F). Overall, these results indicate that *OsSCR106* regulates the expression and RNA splicing of a large number of genes.

### *OsSCR106* is involved in mediating global gene expression and genome-wide splicing under salt stress conditions

**Fig. 8.**
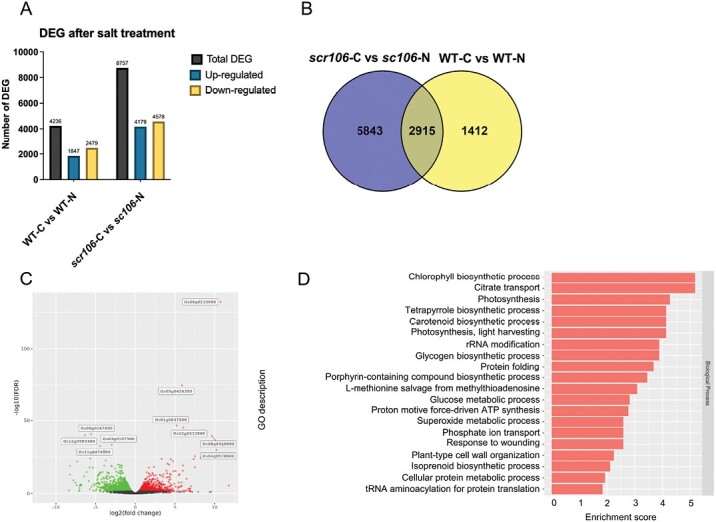
Genome-wide transcriptional effects of the *scr106* mutant under salt stress. (A) Number of differentially expressed genes (DEGs, up- and down-regulated) in *scr106* and the wild type (WT) under salt stress conditions. (B) An overlap between the WT and *scr106*. A large number of the DEGs in the WT show overlap with *scr106.* The majority of the DEGs are uniquely regulated by *OsSCR106* under salt stress conditions. (C) Volcano plot of the DEGs comparing *scr106* and the WT under salt stress. Red dots represent up-regulated genes while green dots denote down-regulated genes. Black dots represent the genes that are not significantly differentially expressed. The gene IDs for the top 10 highly DEGs are shown. (D) GO enrichment analysis of the DEGs regulated by *scr106* under salt stress conditions.

To determine the link between environmental stresses and AS, and to verify the role of *OsSCR106* in regulating stress-induced AS, we performed mRNA sequencing after salt treatment. We found 4236 DEGs in the wild type under salt stress in comparison with plants under control conditions. Among these, 1847 genes were up-regulated and 2479 were down-regulated (Fig. 8A; Supplementary Datasets S3, S4). However, in *scr106*, compared with the wild type, a large number of genes (8757) were differentially expressed under salt treatment. Of these DEGs, 4179 were up-regulated while 4578 were down-regulated. We compared the salt-regulated DEGs in the wild type with the salt-regulated DEGs in *scr106* and found a large overlap of 2915 DEGs; however, 5843 genes were uniquely differentially expressed in the *scr106* mutant (Fig. 8B). This indicates that most of the DEGs observed in the *scr106* mutant are unique and only regulated by *OsSCR106* under salt stress conditions; however, there is also a set of genes that were differentially expressed under salt regardless of the genotype. In a volcano plot, we compared the DEGs of the *scr106* mutant and wild type only after salt treatment. Our analysis showed that unlike control conditions, the top DEGs (based on the highest log-fold expression change) are either up- or down-regulated. Among the top up-regulated genes, *Os06g0215900* is Similar to oxo-phytodienoic acid reductase, *Os01g0647200* is Iron deficiency-inducible peptide, and*Os12g0512800* is cytochrome P450 71E1 (EC 1.14.13.68) (4-hydroxyphenylacetaldehyde oxime monooxygenase). The top down-regulated genes are *Os04g0474800*, Similar to OSIGBa0135C13.7 protein; and *Os01g0167800*, Conserved hypothetical protein (Fig. 8C). The GOEA showed that the global DEGs between the wild type and the *scr106* mutant under salt treatment were enriched in the biological processes of chlorophyll biosynthetic process, citrate transport, photosynthesis, tetrapyrrole biosynthetic process, and carotenoid biosynthetic process (Fig. 8D).

**Fig. 9.**
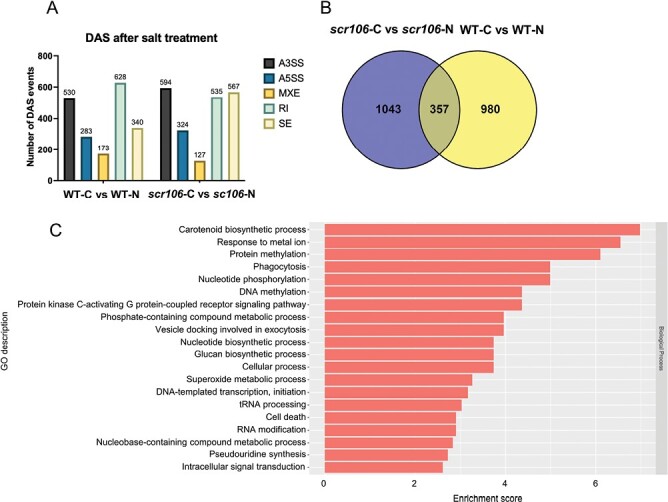
*OsSCR106* alters the pre-mRNA splicing under salt stress. (A) Bar plot showing the number of each type of differential alternative splicing (DAS) event induced by salt treatment in the *scr106* mutant and the wild type (WT). (B) Venn diagram showing the overlap of differential alternative splicing (DAS) genes between *scr106* and WT plants under salt stress conditions. (C) GO enrichment analysis of the DAS genes regulated by *scr106* under salt stress.

To further examine the role of OsSCR106 during pre-mRNA splicing regulation under salt stress, we performed a splicing analysis and identified DAS events for the wild type and *scr106* mutant. All types of AS events were detected, with RIs representing the majority of AS events for the wild type and A3SSs representing the majority of AS events for *scr106* (Fig. 9A; Supplementary Datasets S5, S6). For the wild type, the number of AS events for A3SS was 530; A5SS, 283; MXE, 173; RI, 628; and SE, 340. For *scr106*, the number of AS events for A3SS was 594; A5SS, 324; MXE, 127; RI, 535; and SE, 567. Strikingly, we detected more SE events for *scr106* in comparison with the wild type (Fig. 9A). We compared the DAS genes for the wild type with *scr106* and found that a majority of these DAS genes are unique and only a small number of these genes overlap (Fig. 9B). The GOEA of the global DAS genes between the wild type and the *scr106* mutant under salt treatment showed enrichment for carotenoid biosynthetic process, response to metal ion, protein methylation, phagocytosis, and nucleotide phosphorylation (Fig. 9C). Overall, these results indicate that *SCR106* regulates global gene expression and genome-wide pre-mRNA splicing under salt stress conditions in rice.

**Fig. 10.**
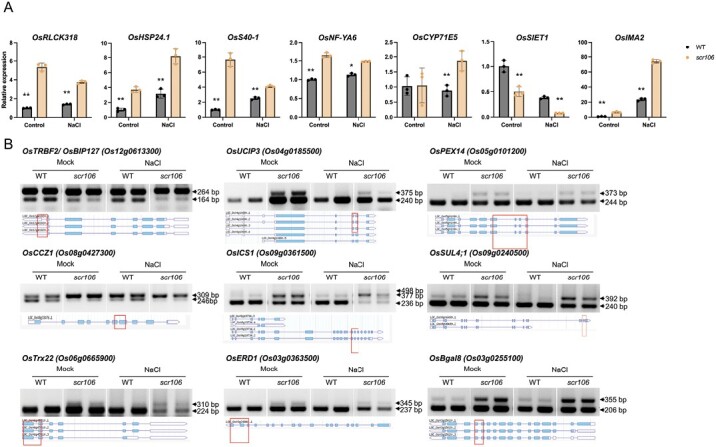
Evaluation of differential expression and differential intron retention (IR) in *scr106* and wild-type (WT) plants. Rice seedlings of the mutant *scr106* and WT were germinated under control and 125 mM NaCl for 1 week. Total RNA extracted from the whole seedling was used for mRNA expression and splicing pattern analysis. (A) Validation of expression of genes detected by RNA-seq. Genes were randomly selected from the lists of DEGs under control or salt treatment. The locus names are *OsRLCK318* (*Os11g0213000*), *OsHSP24.1* (*Os02g0758000*), *OsS40-1* (*Os05g0531100*), *OsNF-YA6* (*Os07g0608200*), *OsCYP71E5* (*Os12g0512800*), *OsSIET1* (*Os03g0107300*), and *OsIMA2* (*Os07g0142100*). Bars represent the mean ±SE of three replicates. *OsActin* was used as an internal control. (Student’s *t*-test; *P<0.05, **P<0.01). (B) Semi-quantitative RT–PCR analysis to validate the IR of randomly selected genes from the lists of DAS genes under control or salt treatment. Arrowheads indicate splicing variants that changed in the *scr106* mutant. The gene structures and retained introns are shown. Red boxes indicate the PCR fragments.

To corroborate our findings from RNA-seq analysis, we investigated the expression profiles of some randomly selected DEGs under control or salt treatment. We germinated the rice seedlings of the wild type and *scr106* mutant for 1 week at 125 mM NaCl and control conditions. The expression levels were tested for the rice genes *OsRLCK318* (*Os11g0213000*), *OsHSP24.1* (*Os02g0758000*), *OsS40-1* (*Os05g0531100*), *OsNF-YA6* (*Os07g0608200*), *OsCYP71E5* (*Os12g0512800*), *OsSIET1* (*Os03g0107300*), and *OsIMA2* (*Os07g0142100*) (Fig. 10A). The expression of *OsCYP71E5* was only induced after salt treatment in the *scr106* mutant. The expression levels of all other genes are changed under salt as well as control conditions in *scr106* compared with the wild type (Fig. 10A). The transcript levels of all genes were significantly up-regulated, except *OsSIET1* for which the expression level was significantly reduced in the *scr106* mutant compared with the wild type under salt and control conditions (Fig. 10A). These results validate our RNA-seq data showing that SCR106 is involved in the transcriptional regulation of a vast set of genes. This is further verified by analysis of the splicing patterns as a set of randomly selected genes whose splicing is inhibited in the *scr106* mutant (Fig. 10B). We found an enrichment of introns in *scr106* compared with wild-type seedlings under salt and control conditions. Interestingly, for some genes such as *OsUCIP3* (*Os04g0185500*), *OsCCZ1* (*Os08g0427300*), and *OsICS1* (*Os09g0361500*), the transcript levels of RIs were even higher under control conditions in the *scr106* mutant. This indicates that *scr106* also regulates the pre-mRNA splicing during the development of rice seedling irrespective of any environmental stress (Fig. 10B). Overall, these results validate our RNA-seq data and confirm that SCR106 is an important splicing factor that regulates the splicing of a vast set of genes.

## Discussion

AS is an important gene regulatory process that generates multiple RNA isoforms from a single gene sequence. In plants, the SR proteins regulate the splice selection under certain developmental and stress conditions to generate specific RNA isoforms. Thus, AS regulated by SR proteins fine-tunes the physiology and metabolism to cope with abiotic stress conditions such as cold, heat, drought, high salinity, and heavy metals. SR proteins are characterized by their ability to interact simultaneously with RNA and other protein components via an RRM and through a domain rich in arginine and serine residues, the RS domain (Shepard and Hertel, 2009; Barta *et al*., 2010). In this study we identified *LOC_Os01g01150* in rice, which fulfills these criteria. We designated it as *OsSCR106* because it groups with the SC-subfamily of SR proteins and the protein has a size of 106 kDa. Based on the amino acid comparison using BLAST, OsSCR106 is a homolog of human SRSF11 and Arabidopsis IRR (At3g23900) (Supplementary Fig. S1). To our surprise, this *OsSCR106* was not considered before for RNA splicing studies in plants. It is important to note that we used BLAST to discover homologs of SRSF11 in Arabidopsis first, which revealed At3g23900, then by BLAST we compared At3g23900 with the rice proteome to get OsSCR106, which could have been missed if SRSF11 had been compared directly with the rice proteome. The SR proteins are prominent components of nuclear speckles, which occur throughout the nucleus, and presumably are the sites for pre-mRNA splicing. (Shepard and Hertel, 2009; Hackmann *et al.*, 2014). For OsSCR106, we observed a localization to nuclear speckles (Fig. 1E), suggesting a role for RNA splicing.

For further studies, we generated the k/o mutant *scr106*. In our analysis, the mutant seedlings showed reduced root and shoot lengths (Fig. 3). In contrast to animals, the loss-of-function mutants of SR proteins in plant are not embryonically lethal. The Arabidopsis *sr45-1* mutant showed many developmental abnormalities including delayed flowering, narrow leaves, disturbed numbers of petals and stamens, and reduced root growth (Ali *et al.*, 2007). Moreover, even the *sr* quadruple mutant (*sr34 sr34a sr34b sr30*), the*rs* quadruple mutant (*rs31 rs31a rs40 rs41*), the *rsz-rs2z* quintuple mutant (*rsz21 rsz22a rsz22 rs2z32 rs2z33*), and the *sc35-scl* quintuple mutant (*scl28 scl30 scl30a scl33 sc35*) were not lethal in Arabidopsis (Yan *et al.*, 2017). The loss of function of SC35 and SCL (SC35-like, SCL28, SCL30, SCL30a, and SCL33) results in pleiotropic changes in development, including serrate rosettes, late flowering, shorter roots, and anomalous phyllotaxis arrangement (Yan *et al.*, 2017). In addition, the overexpression of *AtRSZ33* caused changes in embryo and stomata development, cell expansion and shape, meristem formation, and decreased seed set (Kalyna *et al*., 2003). Collectively, these examples confirm the role that SR proteins play in plant development.

Environmental stress regulation in plants is a complex phenomenon and many of the stress-related genes are susceptible to AS (Reddy *et al.*, 2013; Filichkin *et al.*, 2015; Shang *et al*., 2017; Laloum *et al*., 2018; Rambout *et al*., 2018; Chaudhary *et al.*, 2019; Jabre *et al.*, 2019; Zhang *et al.*, 2019; Zheng *et al.*, 2019; Ganie and Reddy, 2021; Ling *et al*., 2021). The SR or SR-like proteins play a crucial role during the regulation of AS, and thus they orchestrate the plant stress responses (Carvalho *et al.*, 2016; Liu *et al.*, 2016; Yan *et al.*, 2017; de Francisco Amorim *et al.*, 2018; Gu *et al.*, 2018; Albaqami *et al*., 2019; Lu *et al.*, 2020; Muthusamy *et al.*, 2020; Park *et al.*, 2020; Butt *et al.*, 2021, 2022; Laloum *et al.*, 2023). Strikingly, the *scr106* mutant showed hypersensitive phenotypes to salt, ABA, mannitol (Fig. 3), and low-temperature treatments (Fig. 4). This is in line with the previous studies, where *sr45-1*, a loss-of-function, mutant, showed a defective response to glucose and ABA, and was hypersensitive in response to salt stress (Carvalho *et al*., 2010; Albaqami *et al*., 2019). Butt *et al.* (2022) showed that the *rs33* loss-of-function rice mutant was hypersensitive to salt and low-temperature stresses, and that *RS33* regulates pre-mRNA splicing in response to abiotic stresses. Similarly, the *sr34b* mutant displayed a shorter root phenotype in response to cadmium treatment, with higher accumulation of cadmium in the root of *sr34b* in comparison with the wild type (Zhang *et al.*, 2014). Moreover, the loss-of-function mutant *scl30a-1* was hypersensitive under ABA and salt stress during seed germination, whereas *SCL30a* overexpression reduced the sensitivity to ABA and enhanced salt stress tolerance (Laloum *et al.*, 2023). We analyzed the expression of OsSCR106 under different stress conditions and found that only cold treatment significantly affects the expression of OsSCR106 (Supplementary Fig. S4). The SR proteins show different levels of expression and splicing patterns under abiotic stress conditions (Palusa *et al*., 2007; Cruz *et al.*, 2014). Most members of the plant-specific SR genes are affected by stress conditions while the expression of some of the SR genes is not affected. Thus the hypersensitivity response of the *scr106* mutant against salt and ABA stress might be due to the activation/repression of the stress-related genes which are regulated by *OsSCR106*. For the detailed analysis, we retrieved the list of stress-related genes from the RAP database and compared them with DEG and DAS genes. To our surprise, there are a large set of stress-related genes which are affected by *OsSCR106* (Supplementary Tables S2, S3). Under control conditions, 94 stress-related genes were up-regulated and 63 were down-regulated, while under salt stress 177 stress-related genes were up-regulated and 296 were down-regulated (Supplementary Table S2; Supplementary Datasets S7, S8). Similarly, splicing patterns were also changed for stress-related genes; 252 and 256 genes were differentially spliced under control and salt stress conditions, respectively (Supplementary Table S3, Supplementary Datasets S9, S10). These data reinforce the crucial role of *OsSCR106* in plant stress regulation.

In plants, RI is the dominant AS event (Kumar *et al.*, 2022). Surprisingly, in our analysis of the *scr106* mutant, A3SS is a dominant event, under both control and salt stress conditions, which indicates that OsSCR106 is mainly involved in regulating 3'-splice site selection. In a typical splicing reaction, spliceosome assembly begins with the base pairing of the U1 snRNP with the 5ʹ-splice site of the intron in the pre-mRNA strand. This binding is supported by the proteins of U1 snRNP and proteins of the SR family (Wahl *et al*., 2009). This initial assembly also involves the interaction of U2 auxiliary factor (U2AF) with the branch point (BP) and the polypyrimidine tract just downstream of the BP. In eukaryotes, splicing factor 1 (SF1) interacts with the 65 kDa subunit of U2AF (U2AF65) through its C-terminal RRM. The smaller subunit of U2AF35 binds the AG dinucleotide of the 3ʹ-splice site and plays crucial roles in the recognition of the 3ʹ-splice site of an intron (Wu *et al.*, 1999; Wahl *et al*., 2009). In Arabidopsis, a mutation in *AtSF1* induced pleiotropic developmental defects, including early flowering, and showed oversensitivity to ABA treatment (Jang *et al.*, 2014). The high number of mis-spliced events for the 3ʹ-splice site in the *scr106* mutant indicates that OsSCR106 possibly interacts with U2AF35 and regulates the 3ʹ-splice site selection in rice.

Altogether, we identified and revealed the function of *OsSCR106* as a novel splicing factor. *OsSCR106* localizes to the nuclear speckles and regulates the pre-mRNA splicing. *OsSCR106* plays a pivotal role in abiotic stress tolerance in rice. The accurate control over the splicing machinery and its components helps to develop crops adaptable to changing climatic conditions.

## Supplementary data

The following supplementary data are available at *JXB* online.

Fig. S1. Protein alignments of OsSCR106 with Arabidopsis and human homologs.

Fig. S2. Phylogenetic analysis of OsSCR106 with rice SR proteins.

Fig. S3. Protein alignments of OsSCR106 with rice SC-subfamily proteins.

Fig. S4. Gene expression of OsSCR106 under different stress conditions.

Table S1. List of sequences used in this study.

Table S2. Number of stress-related DEG per comparison.

Table S3. Number and type of stress-related AS events per comparison.

Dataset S1. List of DEG SR-C_vs_WT-C.

Dataset S2. List of DAS SR-C_vs_WT-C.

Dataset S3. List of DEG WT-N_vs_WT-C.

Dataset S4. List of DEG SR-N_vs_SR-C.

Dataset S5. List of DAS WT-N_vs_WT-C.

Dataset S6. List of DAS SR-N_vs_SR-C.

Dataset S7. List of stress-related DEGs under control.

Dataset S8. List of stress-related DEGs under salt.

Dataset S9. List of stress-related DAS events under control.

Dataset S10. List of stress-related DAS events under salt.

erad433_suppl_Supplementary_Tables_S1-S3_Figures_S1-S4

erad433_suppl_Supplementary_Datasets_S1

erad433_suppl_Supplementary_Datasets_S2

erad433_suppl_Supplementary_Datasets_S3

erad433_suppl_Supplementary_Datasets_S4

erad433_suppl_Supplementary_Datasets_S5

erad433_suppl_Supplementary_Datasets_S6

erad433_suppl_Supplementary_Datasets_S7

erad433_suppl_Supplementary_Datasets_S8

erad433_suppl_Supplementary_Datasets_S9

erad433_suppl_Supplementary_Datasets_S10

## Data Availability

All data supporting the findings of this study are available within the paper and its supplementary data published online. RNA-seq data that support the findings of this study have been deposited in the NCBI Bioproject database. The RAW data and processed data files were deposited in GEO (https://www.ncbi.nlm.nih.gov/geo/) under record GSE232160.
