# Supplementary material for: SCR106 splicing factor modulates abiotic stress responses by maintaining RNA splicing in rice
Source: J Exp Bot. 2023 Oct 31;75(3):802–18. doi: 10.1093/jxb/erad433 (PMC10837019; doi:10.1093/jxb/erad433)
Supplement: erad433_suppl_Supplementary_Tables_S1-S3_Figures_S1-S4 [file erad433_suppl_supplementary_tables_s1-s3_figures_s1-s4.pdf]

SCR106 splicing factor modulates abiotic stress responses by maintaining the RNA splicing in rice

Abdulrahman Alhabsi[1], Haroon Butt[1], Gwendolyn K. Kirschner[2], Ikram Blilou[2], and Magdy M. Mahfouz[1,*]

[1]Laboratory for Genome Engineering and Synthetic Biology, Division of Biological Sciences, 4700 King Abdullah University of Science and Technology, Thuwal 23955-6900, Saudi Arabia.
[2]Laboratory of Plant Cell and Developmental Biology, Division of Biological Sciences, 4700 King Abdullah University of Science and Technology, Thuwal 23955-6900, Saudi Arabia.

*Correspondence: Magdy M. Mahfouz (magdy.mahfouz@kaust.edu.sa)

Highlight

OsSCR106, a splicing factor that is related to the SC-subfamily of SR proteins, plays an essential role in abiotic stress tolerance by controlling alternative gene splicing in rice.

Abstract

Plants employ sophisticated molecular machinery to fine-tune their responses to growth, developmental, and stress cues. Gene expression influences the plant's cellular responses through regulatory processes like transcription and splicing. Pre-mRNA is alternatively spliced to increase the genome coding potential and further regulate the expression. Serine/Arginine-rich (SR) proteins, a family of pre-mRNA splicing factors, recognize splicing *cis*-elements and regulate both constitutive and alternative splicing. Several studies reported SR protein genes in the rice genome, subdivided into six subfamilies based on their domain structures. Here, we identified a new splicing factor in rice with RNA recognition motif (RRM) and SR-dipeptides and is related to the SR proteins, subfamily SC. OsSCR106 regulates pre-mRNA splicing under abiotic stress conditions. OsSCR106 localizes to the nuclear speckles, a major site for pre-mRNA splicing in the cell. Loss-of-function *scr106* mutant is hypersensitive to salt, ABA, and low-temperature stress and harbors developmental abnormality indicated by the shorter length of shoot and root. The hypersensitivity to stress phenotypes was rescued by complementation using OsSCR106 fused behind its endogenous promoter. Global gene expression and genome-wide splicing analysis in wild-type and *scr106* seedlings revealed that OsSCR106 regulates its targets, presumably through regulating the alternative 3´ splice site. Under salt stress conditions, we identified multiple splice isoforms regulated by OsSCR106. Collectively, our results suggest *OsSCR106* is an important splicing factor that plays a crucial role in accurate pre-mRNA splicing and regulates abiotic stress responses in plants.

Keywords: Pre-mRNA Splicing, Alternative Splicing, SR Proteins, Genome Engineering, abiotic stress

Introduction

Plants adapt to stresses with different molecular mechanisms that mainly regulate gene expression under stressful conditions (Reddy *et al.*, 2013). Pre-mRNA splicing is one of the processes that regulate gene expression under abiotic stress conditions and enhances the plasticity of the genome. RNA splicing eliminates non-coding regions (introns) in constitutive splicing, producing a single transcript (Will and Luhrmann, 2011). However, alternative splicing (AS), common in plants, produces multiple isoforms from a single gene, increasing transcriptome and potential proteome complexity. AS generates transcripts with retained intron (RI), skipped exon (ES), alternative 5′ splice site (A5SS), alternative 3′ splice site (A3SS), or mutually exclusive exons (MXE) (Ganie and Reddy, 2021; Laloum, Martin and Duque, 2018; Reddy *et al.*, 2013)

RNA splicing is carried out by the spliceosome, a large ribonucleoprotein complex. The U2-type spliceosome is the primary complex with five uridine-rich small nuclear ribonucleoprotein particles (snRNPs), U1, U2, U5, U4/U6, and many non-SnRNPs (Gehring and Roignant, 2021; Marasco and Kornblihtt, 2022; Shi, 2017; Will and Luhrmann, 2011). For RNA splicing, spliceosomal components assemble to bind respective pre-mRNA and remove introns marked by 5′ and 3′ splice sites, branch site, and polypyrimidine tract (Keren, Lev-Maor and Ast, 2010). In plants, chemical or genetic interference of spliceosome activity can inhibit the splicing leading to cell death (AlShareef *et al.*, 2017; Butt *et al.*, 2021; Butt *et al.*, 2019a; Ling *et al.*, 2017). Many factors facilitate the spliceosome assembly at the pre-mRNA, including Serine/Arginine-rich (SR) proteins, which recognize and bind splicing regulatory sites in pre-mRNA, regulating the splicing (Di Zhang, 2020; Gehring and Roignant, 2021; Marasco and Kornblihtt, 2022; Morton *et al.*, 2019; Shi, 2017).

The SR protein family is a conserved RNA-binding protein family among metazoans, plants, fungi, and protozoa (Plass *et al.*, 2008; Richardson *et al.*, 2011). Plant SR proteins were discovered by investigating sequence homology to mammalian SR proteins or interactions with U1 snRNP (70K, (U1-70K) (Birney, Kumar and Krainer, 1993; Golovkin and Reddy, 1998; Lazar *et al.*, 1995). There are two SR proteins in

fission yeast (Barbosa-Morais, Carmo-Fonseca and Aparicio, 2006), 12 in humans (Manley and Krainer, 2010), 18 in Arabidopsis, and 22 in rice (Richardson *et al.*, 2011). They have also been identified in algae, maize, wheat, soybean, sorghum, grape, and other plants (Chen *et al.*, 2019; Di Zhang, 2020; Rauch *et al.*, 2014). An SR protein contains either a single or double N-terminal RNA recognition motif (RRM) and a C-terminal Arginine/Serine-rich (RS) domain for protein-protein interactions (Barta, Kalyna and Reddy, 2010; Di Zhang, 2020). The phosphorylation state of residues in RS domains can influence protein interaction, subcellular localization, and splicing activity of SR proteins (Stamm, 2008; Xiang *et al.*, 2013; Zhou and Fu, 2013). SR proteins are classified into six subfamilies, among which SR, SC, and RSZ are conserved between humans and plants, whereas RS, SCL, and RS2Z are plant-specific (Barta, Kalyna and Reddy, 2010; Morton *et al.*, 2019; Reddy and Shad Ali, 2011). Interestingly, the wide spread of SR proteins in higher eukaryotes corresponds with elevated AS complexity, suggesting their essential role in regulating complex splicing events (Busch and Hertel, 2012). Moreover, SR proteins are employed in many post-transcriptional processes, including mRNA movement, stability, and translation (Jeong, 2017). SR proteins are highly dynamic and mainly localized to nuclear speckles (Caceres *et al.*, 1997), in which splicing factors are stored before recruitment to splicing events in the nucleoplasm (Galganski, Urbanek and Krzyzosiak, 2017; Misteli, Caceres and Spector, 1997). Many SR proteins shuttle back and forth between the nucleus and cytoplasm (Caceres, Screaton and Krainer, 1998).

Investigations on plant SR proteins have emerged slower than in animals, and most of the studies have linked plant AS to physiological and stress responses (Carvalho, Feijao and Duque, 2013; Laloum, Martin and Duque, 2018; Staiger and Brown, 2013). The expression pattern of SR genes changed in response to the development or stress conditions, indicating possible involvement in regulating these conditions (Duque, 2011; Filichkin *et al.*, 2015; Jiang *et al.*, 2017; Reddy and Shad Ali, 2011). The *Arabidopsis* loss-of-function mutant *sr45-1* has provided insights toward understanding the physiological role of plant SR proteins (Di Zhang, 2020). *sr45-1* exhibited developmental abnormalities (Ali *et al.*, 2007a) and was sensitive to sugar, ABA, and salt treatment

(Albaqami, Laluk and Reddy, 2019; Carvalho, Carvalho and Duque, 2010; Carvalho *et al.*, 2016). The *SR45* produced two isoforms; the SR45.1 isoform was involved in flower development, whereas SR45.2 was involved in root growth (Albaqami, Laluk and Reddy, 2019; Zhang and Mount, 2009). The loss-of-function mutants of other members of the SR family, such as *rs40, rs41,* and *scl30a*, showed hypersensitivity to salt and abscisic acid (ABA) stresses in *Arabidopsis thaliana (Chen et al., 2013; Laloum et al., 2021).*

In rice, abiotic stresses such as ABA, NaCl, heat, and cold induce AS of SR transcripts (Zhang *et al.*, 2013). SR proteins maintain nutrient homeostasis, such as Phosphate remobilization by SR40, SCL25, and SCL57 in rice shoots (Dong *et al.*, 2018). Recently, functional investigation analysis showed that *rs33* loss-of-function rice mutant was hypersensitive to salt and low-temperature stresses, and that *RS33* regulates pre-mRNA splicing in response to abiotic stresses (Butt *et al.*, 2019b; Butt, 2022). In contrast, no morphological changes were observed in rice after overexpressing several SR genes individually, including *SR32*, *SR33a*, *SR33*, *SCL26*, *RSZ23*, *RS2Z36*, and *RS2Z37*, although lines overexpressing *RS29* and *RS33* were lethal as they could not be recovered (Isshiki, Tsumoto and Shimamoto, 2006). Moreover, increased expression of *SCL30* compromised salt, drought, and low temperature tolerance in rice (Wu *et al.*, 2022). In addition, single and multiplex mutants of SR protein genes were created using the CRISPR/Cas system and may be utilized to understand the role of SR proteins in plant development and stress response (Butt *et al.*, 2019b). Overall, the above studies indicate that SR proteins regulate the pre-mRNA splicing under abiotic stress conditions; thus, accurate and efficient regulation of these splicing factors may increase the adaptation to abiotic stress. However, most of these proteins are not well characterized, and also the role of a single SR protein and the splicing events they regulate is not yet clear.

In the present study, we characterized a novel splicing factor termed *OsSCR106* in rice. The phylogenetic analysis showed that this protein is rich in SR dipeptides and contains an RRM domain at its N-terminal. Expression analysis showed that *OsSCR106* is expressed in seed endosperm, leaves, and vascular tissues. The *OsSCR106:EGFP*

fusion under *UBIQUITIN* promoter localized to the nuclear speckles. We investigated the function of *SCR106* via targeted mutagenesis using the CRISPR/Cas9 system. The *scr106* mutant, like other SR proteins, is hypersensitive to abiotic stress conditions like salt and low temperature. These hypersensitive phenotypes were rescued via the expression of *OsSCR106* cDNA. The RNA-seq analysis identified a subset of genes involved in salt stress responses, which are regulated by *OsSCR106*. Our results identified OsSCR106 as a splicing factor and provide its critical role in regulating the AS and determining stress responses.

Materials and Methods

Plant materials

Oryza sativa L. ssp. *japonica* cv. Nipponbare was used for all experiments. All plants were grown in greenhouse at 28°C. Plants were supplemented with Hoagland solution biweekly.

Vector construction

For targeted mutagenesis, the expression of Cas9 was driven by *OsUbiquitin,* and the sgRNA was expressed under the *OsU3* promoter. The sgRNAs were designed to target the first exon of the *OsSCR106* (*LOC_Os01g01150; Os01g0101600*) in rice. The *pRGEB32* plasmid (Butt *et al.*, 2019b; Xie, Minkenberg and Yang, 2015) was digested with *Bsa*I, sgRNAs were synthesized as oligonucleotides with *Bsa*I overhangs, GGCA in the forward oligonucleotides and AAAC in reverse. The oligonucleotides were annealed and ligated in the *Bsa*I-digested vector.

To generate the overexpression construct tagged with GFP, *pUBI::OsSCR106-EGFP*, we amplified the full-length coding sequence of *OsSCR106* and cloned it into the binary vector pENTR™/D-TOPO™ (Thermo Fisher Scientific) according to the manufacturer's instructions. The GFP sequence is amplified using primers EcoRI_GFP_F and

KpnI_GFP_R to clone inframe with *OsSCR106.* Using gateway cloning, an LR reaction was performed to transfer OsSCR106-EGFP into the destination vector pRGEB32.

To generate the promoter analysis construct, *pOsSCR106::EGFP-GUS*, we amplified ~ 2.5 kb upstream and 45 bp downstream of the start codon (ATG) of *OsSCR106*. The PCR products were then cloned into the binary vector pENTR™/D-TOPO™ according to the manufacturer's instructions. Then we performed an LR reaction to transfer *pOsSCR106* inframe with EGFP-GUS into the destination vector pKGWSF7 (Karimi, Inze and Depicker, 2002).

To generate the complementation construct, *pOsSCR106::OsSCR106-EGFP*, we amplified and cloned the *pOsSCR106* by restriction digestion into pRGEB32 by replacing the *OsUbiquitin* promoter sequence. *OsSCR106-EGFP* was amplified from the vector *pUBI::OsSCR106-EGFP* and cloned downstream *pOsSCR106* in the pRGEB32 destination vector.

Rice transformation

Agrobacterium-mediated rice transformation was performed as described previously (Butt *et al.*, 2019b; Hiei and Komari, 2008). The *Agrobacterium tumefaciens* strain EHA105 was used for all transformations. For pRGEB32 vectors 50 mg/L of Hygromycin was used for selection and regeneration. For pKGWSF7 vector, 150 mg/L of G418 was used for selection and 100 mg/L of G418 was used for regeneration.

Genotyping of the OsSCR106 mutant plants

DNA was extracted from rice leaves frozen in liquid nitrogen during collection. PCR was performed using the gene-specific primers to amplify the gRNA targeted region. PCR product size was confirmed by gel electrophoresis and gel purified then cloned into pJET1.2 blunt vector using CloneJET PCR Cloning Kit (K1231). The clones were then Sanger sequenced to analyze the mutations.

Phenotypic analysis of mutants and complementation lines under abiotic stress conditions

We used homozygous k/o, Cas9 free, and T2 or T3 generations for all phenotypic analysis. Freshly harvested seeds of each genotype were used for stress tolerance assays. The sterilized seeds were transferred to ½-strength Murashige and Skoog (½ MS) solid medium supplemented with and without 100 mM NaCl, 125 mM NaCl, 2 µM ABA, 5 µM ABA, 100 mM mannitol, and 200 mM mannitol. The plates were sealed and incubated in a plant growth chamber maintained at 28/26°C, 16/8 h day/night photoperiod. The seedlings were allowed to grow vertically for seven days. The control conditions are without NaCl. ABA, and mannitol.

To test the low-temperature tolerance of these genotypes, petri plates were transferred to different growth chambers maintained at the indicated temperatures for a specified time. The seeds initially subjected to the low-temperature treatment of 16 and 4°C were transferred to 28/26°C after 14 days, for five days. The seeds were allowed to grow for 12 days at 28/26°C, 22/20°C, or 18/15°C. The control is normal growth conditions at 28/26°C.

Histochemical analysis of *OsSCR106* gene expression

Transgenic seedlings or plant organs carrying GUS reporter constructs were harvested, dipped in 3 ml of GUS staining buffer in a 6-well culture plate and incubated at 37°C. If necessary, vacuum was applied for 10 min at RT to facilitate buffer uptake. When staining was complete, the samples were rinsed with dH_2O and de-stained by shaking overnight at RT in 70% EtOH. The plant material was rehydrated by successive 15 min incubation in 40%, 20% and 10% (v/v) EtOH followed by an overnight incubation in 5% (v/v) EtOH/ 25% (v/v) glycerol.

Subcellular localization analysis

Roots of *pUBI::OsSCR106-EGFP* and *pOsSCR106::OsSCR106-EGFP* fixed in 4% para-formaldehyde in PBS for 2 hours, and then washed with dH_2O and incubated in

ClearSee solution (Kurihara *et al.*, 2015) for ~2 weeks and then imaged with the Zeiss LSM710 inverted microscope.

RNA isolation and sequencing

Total RNA was extracted from seven-day-old rice seedlings grown in ½ MS media with or without 125 mM NaCl using the Direct-zol RNA MiniPrep Plus kit (Zymo Research). We followed the manufacturer's recommendations. RNA was quantified using a Nanodrop, and RNA quality was examined using a 2100 Bioanalyzer (Agilent Technologies). High-quality RNA samples with RNA integrity number (RIN) ≥7.0 were selected for library construction. The RNA-seq libraries were constructed using the TruSeq mRNA stranded kit following the standard protocol and sequenced on the Nova-seq platform to generate high-quality paired-end reads.

Analysis of RNA-seq data and gene functional classification

RNA-seq data analysis was performed by Sequentia Biotech®. The dataset included RNA-seq data from 12 samples belonging to two genotypes (*scr106* and WT), two experimental conditions (salt stress - N, and control - C) and three biological replicates. The quality of the reads was assessed with the software FASTQC, then a trimming step was performed in order to remove adapters and low-quality bases from the reads. The following parameters were used: minimum length was set to 35 bp and the quality score to 25. The software TRIMMOMATIC was used for this scope. On average, 43.5 million of filtered reads were obtained per sample. The high-quality reads were aligned against the *Oryza sativa* cv. *Nipponbare* genome (IRGSP 1.0) with STAR aligner (version 2.7.10a). On average, 88.95 % of the reads could be mapped uniquely on the genome. FeatureCounts (version 2.0.0) was used to calculate gene expression values as raw fragment counts. In addition, a normalization was applied to the raw fragment counts by using the Trimmed Mean of M -values (TMM) and the Fragments Per Kilobase Million (FPKM) normalization. All the statistical analyses were performed with R with the packages HTSFilter and edgeR.

The edgeR package determines differential expression using empirical Bayes estimation and exact tests based on a negative binomial model. The genes showing an FDR less or equal than 0.05 were considered to be statistically significant. For the significantly differentially expressed genes a Gene Ontology Enrichment Analysis (GOEA) was performed to identify the most enriched Gene Ontology (GO) categories across the down- and up-regulated genes.

In addition to the differential expression analysis of the genes, a differential splicing analysis was performed using the software RMATS (version 4.1.2). Only the differential events supported by an FDR <=0.05 and an absolute IncLevelDifference > 0.1 were considered.

RT-PCR and RT-qPCR

For reverse-transcription PCR (RT-PCR), DNA digestion of total RNA samples was performed using an RNase-Free DNase Set (Invitrogen cat. No. 18068-015) following the manufacturer's protocol. The total RNA was reverse transcribed using a SuperScript First-Strand Synthesis System (Invitrogen) to generate cDNA. PCR conditions were: initial denaturation at 95°C for 2 minutes, then 40 cycles of 95°C for 30 seconds, 55°C for 30 seconds, and 72°C for 60 seconds, then final elongation at 72°C for 5 minutes.

The RT-qPCR was performed using the 50 ng RNA in 10 µl final reaction volume. The iTaq™ Universal SYBR® Green One-Step Kit (Bio-Rad, cat. no. 172-5150) was used with the following manufacturer's protocol conditions: reverse transcription 50 °C for 10 min, followed by 40 cycles of 95 °C for 15 s, 60 °C for 1 min, melt-curve analysis 65-95 °C, 0.5 °C increment. The gene expression data were normalized using the $2^{-\Delta\Delta C_T}$ method with three biological replicates. The expression values were normalized with respect to the values of *OsActin* (*LOC_Os03g50885*). All expression analysis was performed on three biological replicates. Primers used for RT-PCR and RT-qPCR are listed in table S3.

Results

OsSCR106 is a homolog of Human SRSF11 and contains plant SR protein characteristics

In rice, only 22 SR proteins have been identified (Barta, Kalyna and Reddy, 2010; Morton *et al.*, 2019; Richardson *et al.*, 2011). To further identify proteins that contain RS dipeptides, we used the Basic Local Alignment Search Tool (BLAST) to compare the amino acid sequence of Human splicing factor SRSF11 against the Arabidopsis proteome and obtained an orthologue, At3g23900, with 32% identity. *At3g23900* has been recently characterized as an immunoregulatory RNA-binding (IRR) protein with three domains, including a zinc finger, RRM, and an RS dipeptide (Dressano *et al.*, 2020). IRR was involved in the negative regulation of immune response in maize and Arabidopsis (Dressano *et al.*, 2020). To investigate orthologues in rice, we then used BLAST to compare the amino acid sequence of At3g23900 against the rice proteome and identified a putative, uncharacterized orthologue, LOC_Os01g01150, with almost 60% identity. We aligned the amino acid sequences of Human SRSF11, Arabidopsis At3g23900, and rice LOC_Os01g01150 to investigate conserved amino acid regions. The results suggest a conserved N-terminal region with RNA Recognition Motif (RRM) and a C-terminal region with many serine/arginine (SR) dipeptides (**Fig. S1**). We generated a phylogenetic tree to investigate the evolutionary relationship among the 22 rice SR genes, including LOC_Os01g01150. The results indicated that LOC_Os01g01150 is closely related to SC subfamily members (**Fig. S2**). Then we aligned the protein sequence of LOC_Os01g01150 with rice SC-subfamily proteins and found this new orthologue has an RRM and an RS domain like all SC subfamily members (**Fig. S3**). The RS domain in LOC_Os01g01150 contains at least 350 amino acids with almost 25% SR or RS dipeptides. In addition, the LOC_Os01g01150 protein has an N-terminus Filamin and Zinc-finger domains which might have some additional functions (**Fig. 1A**). Based on the similarity to domain structure of SR subfamily SC and with molecular weight of 106 kDa, we propose the name *OsSCR106* (*SC-Related106*) for the locus *LOC_Os01g01150*.

***OsSCR106* is expressed in different rice tissues, and the protein localizes to nuclear speckles**

To study the expression pattern of *OsSCR106* in different rice organs, we fused the β-glucuronidase (*GUS*) reporter under the 2.5 kb promoter sequence of *OsSCR106,* and stabily transformed rice calli using agrobacteria (**Fig. 1B**). The histochemical analysis showed GUS activity in the seed endosperm, the leaf sheath, and leaves (**Fig. 1C**). In the root, GUS activity was detected in the vascular cylinders of both primary and lateral roots.

The major splicing activity is performed in the nuclear speckles of the cell (Shepard and Hertel, 2009). To analyze the localization of OsSCR106 in planta, we fused the 2.934 kb CDS of *OsSCR106* to the EGFP and expressed it under the rice *UBIQUITIN* promoter (*pUBI*), stably in rice (**Fig. 1D**). OsSCR106:EGFP localized to nuclear speckles in rice roots (**Fig. 1E, Fig. 1F**). Broad expression of *OsSCR106* promoter and OsSCR106 localization to nuclear speckles indicates a possible role during the pre-mRNA splicing regulation in rice.

Targeted mutagenesis of the rice *OsSCR106* locus

To further investigate the role of *OsSCR106* in rice, we targeted the *OsSCR106* locus in rice using the CRISPR/Cas9 system. The *OsSCR106* locus has three exons, and we designed two different sgRNAs to target the first exon (**Fig. 2A**). These sgRNAs were cloned under the *U3* promoter, and Cas9 expression was driven by the *OsUbiquitin* promoter. We delivered the plasmid in rice callus using the Agrobacterium-mediated transformation method. We recovered the transgenic lines and analyzed the targeted regions via Sanger sequencing. We identified several mutants in the T_0 lines and continued with homologous knockouts (k/o) that contained a premature stop codon (PTC) downstream of the target site (**Fig. 2B**). For k/o line *scr106-8*, the insertions of 1 bp (T) at target site 1 (TS1) changes the downstream protein sequence and causes a PTC after 143 amino acids. Similarly, the k/o *scr106-55* with a deletion of 1 bp (A or T) at target site 2 (TS2) changes the downstream protein sequence and causes PTC after 25 amino acids (**Fig. 2B**). We continued with k/o line *scr106-8* (*scr106*) for the future experiments.

The *scr106* mutant is hypersensitive to abiotic stress conditions

Plant SR proteins are critical modulators in abiotic stress responses, and most of the SR pre-mRNAs are alternatively spliced under abiotic stress conditions (Zhang *et al.*, 2013). The SR mutants were shown to be hypersensitive to abiotic stresses such as salt, ABA, and cold (Albaqami, Laluk and Reddy, 2019; Butt, 2022; Carvalho, Carvalho and Duque, 2010). To investigate the biological function of *OsSCR106* in rice under abiotic stress conditions, we germinated WT and *scr106* mutant seeds on a growth medium supplemented with different stress treatments (**Figure 3**). Under control conditions, the *scr106* mutant exhibited slightly shorter root and shoot lengths than WT seedlings seven days after germination. (**Fig. 3A, Fig. 3B, Fig. 3C**). This might indicate that OsSCR106 regulates the growth and development of rice plants.

We next tested the salt stress response of our mutant as compared to WT. For this, we used two different concentration of 100 mM NaCl and 125 mM NaCl (**Fig. 3D, Fig. 3E, Fig. 3F**). Root and shoot length of the mutants was significantly reduced compared to the WT seedlings at 100 mM NaCl treatment, and even stronger at 125 mM NaCl treatment, indicating that *oscr106* is hypersensitive to salt stress (**Fig. 3D, Fig. 3E, Fig. 3F**). We further tested the growth response of WT and *scr106* mutant under ABA stress treatment (**Fig. 3G, Fig. 3H, Fig. 3I**). At 2 μM ABA treatment the root and shoot length of *scr106* mutant seedlings were significantly shorter as compared to WT seedlings, and growth of *scr106* seedlings was almost ceased at concentrations of 5 μM ABA (**Fig. 3G, Fig. 3H, Fig. 3I**). For osmotic stress treatments we used mannitol at two different concentrations. Mannitol has little impact on shoot length in *scr106* mutant as compared to WT (**Fig. 3J, Fig. 3K, Fig. 3L**). However, the root length was affected at 100 mM mannitol and severely reduced at 200 mM mannitol as compared to WT.

It has been recently shown that alternative splicing impacts the cold responses in plants, and mutants of some splicing factors are hypersensitive to low-temperature treatment (Butt, 2022; Calixto *et al.*, 2018; Lu *et al.*, 2020). To test if OsSCR106 regulates growth under low temperatures in rice, we germinated WT and *scr106* mutant under various low-temperature treatments, including 22 ºC and 18 ºC continuously for 12 days. We observed that the continuous low temperatures of 22 ºC and 18 ºC significantly affected the root and shoot growth in *scr106* mutant compared to WT

seedlings. The growth at 18 ºC is severely inhibited in *scr106* mutant compared to WT seedlings (**Fig. 4A, Fig. 4B, Fig. 4C**). To test the recovery after low-temperature treatment, we used 16 ºC and 4 ºC for 14-days and recovered for 5 days. With the decrease in the temperature treatment, the severity of the growth inhibition increased in *scr106* mutant as compared to WT seedlings (**Fig. 4A, Fig. 4B, Fig. 4C**). The treatment at 4 ºC for 14-days has the highest impact on the recovery of the seedlings. The root and shoot lengths of *scr106* are severely affected compared to WT at 4 ºC (**Fig. 4A, Fig. 4B, Fig. 4C**).

Taken together, these results suggest that *OsSCR106* is essential for abiotic stress regulation in rice.

Expression of *OsSCR106* cDNA in the *scr106* mutant complements its function

To confirm that the loss-of-function mutation is responsible for the developmental abnormalities, and hypersensitivity to stress phenotype, we reintroduced the full-length isoform of *OsSCR106* as cDNA driven by the endogenous promoter in the mutant background. We recovered the transgenic plants and performed analysis using two independent complementation lines in the progeny. Under control conditions, the shoot and root of complementation lines showed full restoration of the WT phenotype (**Fig. 5A, Fig. 5B, Fig. 5C**). Furthermore, the root and shoot length of the complementation lines were fully restored to WT levels under salt and ABA treatment, as well as under low temperature (**Fig. 6A, Fig. 6B, Fig. 6C**). Overall, these results confirm that the hypersensitive reactions to abiotic stress treatments in the *scr106* mutant are caused by the mutation in OsSCR106.

OsSCR106 regulates gene expression and controls the RNA splicing of large number of genes in rice

To understand the role of OsSCR106 in genome-wide pre-mRNA splicing, we performed RNA-seq analysis of seven-day-old seedlings of *scr106* mutant and WT. Comparing the WT transcriptome to the *scr106* mutant identified 1180 differentially

expressed genes (DEGs) between the WT and *scr106*. Among these, 723 genes were upregulated, and 457 genes were downregulated (**Fig. 7A**). The top10 DEGs (based on the highest logfold expression change) were upregulated genes and included *Os06g0215900*, annotated as Similar to Oxo-phytodienoic acid reductase, *Os11g0113500* annotated as retrotransposon gene 1, *Os04g0352400* is FK506-binding protein, Peptidyl-prolyl cis/trans isomerase, Chilling tolerance, *Os11g0213000* is Similar to Protein kinase domain containing protein, expressed, and *Os07g0432333* is Similar to Thionin-like peptide (**Fig. 7B**). The Gene Ontology (GO) enrichment analysis showed that the global DEGs between the WT and the *scr106* mutant under control condition were enriched for the biological processes related to defense response to fungus, defense response to bacterium, response to water, pentose-phosphate shunt, and protein metabolic process (**Fig. 7C**).

To further examine the global defects in pre-mRNA splicing regulated by OsSCR106, we compared the *scr106* and WT and identified differentially alternatively spliced (DAS) events. All types of events, such as A3SS, A5SS, MXE, RI, and SE, were detected. Among these, the alternative 3′ splice site (A3SS) and the retained introns (RI) were the highest differentially alternatively spliced DAS events between the WT and *scr106*, with 1699 A3′SS and 1409 RI events, respectively (**Fig. 7D**). The least number of events were detected for MXE, with 198 events. The GO enrichment analysis showed that the global DAS events between the WT and the *scr106* mutant under control conditions were enriched in Carotenoid biosynthetic process, Branched-chain amino acid metabolic process, Phosphate-containing compound metabolic process, Glutathione biosynthetic process, and Pentose-phosphate shunt (**Fig. 7E**). We then compared the DAS genes with DEGs and found that most of the DEGs were unique, and very few of them were alternatively spliced, uncoupling the transcription from AS regulation (**Fig. 7F**). Overall, these results indicate that *OsSCR106* regulates the expression and RNA splicing of a large number of genes.

***OsSCR106* is involved in mediating global gene expression and genome-wide splicing under salt stress conditions**

To find out the link between environmental stresses and AS and to verify the role of *OsSCR106* in regulating stress-induced AS, we performed mRNA sequencing after salt treatment. We found 4236 DEGs in WT under salt stress in comparison to plant under control conditions. Among these, 1847 genes were up-regulated and 2479 were down-regulated (**Fig. 8A**). However, in *scr106*, compared to WT a large number of genes 8757 were differentially expressed under salt treatment. Among these DEGs, 4179 were up-regulated while 4578 are down-regulated. We compared the salt-regulated DEGs in WT with the salt-regulated DEGs in *scr106* and found a large overlap of 2915 DEGs, however, 5843 genes were uniquely differentially expressed in the *scr106* mutant (**Fig. 8B**). This indicates that most of the DEGs observed in the *scr106* mutant are unique and only regulated by *OsSCR106* under salt stress conditions, however there is also a set of genes that were differentially expressed under salt regardless of the genotype. In volcano plot we compared the DEGs of *scr106* mutant and WT only after salt treatment. Our analysis showed that unlike control conditions top DEGs (based on the highest logfold expression change) are either up- or down-regulated. Among the top up-regulated genes *Os06g0215900* is Similar to Oxo-phytodienoic acid reductase, and *Os01g0647200* is Iron deficiency-inducible peptide, *Os12g0512800* is Cytochrome P450 71E1 (EC 1.14.13.68) (4-hydroxyphenylacetaldehyde oxime monooxygenase). The top down-regulated genes are *Os04g0474800* is Similar to OSIGBa0135C13.7 protein and *Os01g0167800* is Conserved hypothetical protein (**Fig. 8C**). The GO enrichment analysis showed that the global DEGs between the WT and the *scr106* mutant under salt treatment were enriched in the biological processes of Chlorophyll biosynthetic process, Citrate transport, Photosynthesis, Tetrapyrrole biosynthetic process, and Carotenoid biosynthetic process (**Fig. 8D**).

To further examine the role of OsSCR106 during pre-mRNA splicing regulation under salt stress, we performed a splicing analysis and identified DAS events for WT and *scr106* mutant. All types of AS events were detected, with RI representing the majority of AS events for WT and A3SS representing the majority of AS events for *scr106* (**Fig. 9A**). For WT, the number of AS events for A3SS, 530; A5SS, 283; MXE, 173; RI, 628; and SE 340. For *scr106* the number of AS events for A3SS, 594; A5SS, 324; MXE, 127;

RI, 535; and SE, 567. Strikingly, we detected more SE events for *scr106* in comparison to WT (**Fig. 9A**). We compared the DAS genes for WT with *scr106* and found that a majority of these DAS genes are unique and only a small number of these genes overlap (**Fig. 9B**). The GO enrichment analysis of the global DAS genes between the WT and the *scr106* mutant under salt treatment showed the enrichment for Carotenoid biosynthetic process, Response to metal ion, Protein methylation, Phagocytosis, and Nucleotide phosphorylation (**Fig. 9C**). Overall, these results indicate that *SCR106* regulates global gene expression and genome-wide pre-mRNA splicing under salt stress conditions in rice.

To corroborate our findings from RNA-seq analysis, we investigated the expression profiles of some randomly selected differentially expressed genes under control- or salt treatment. We germinated the rice seedlings of the WT and *scr106* mutant for one-week at 125 mM NaCl and control conditions. The expression levels were tested for the rice genes *OsRLCK318* (*Os11g0213000*), *OsHSP24.1* (*Os02g0758000*), *OsS40-1* (*Os05g0531100*), *OsNF-YA6* (*Os07g0608200*), *OsCYP71E5* (*Os12g0512800*), *OsSIET1* (*Os03g0107300*), and *OsIMA2* (*Os07g0142100*) (**Fig. 10A**). The expression of *OsCYP71E5* was only induced after salt treatment in the *scr106* mutant. The expression levels of all other genes are changed under salt as well as control conditions in *scr106* compared to WT (**Fig. 10A**). The transcript levels of all genes were significantly upregulated except *OsSIET1* for which the expression level was significantly reduced in *scr106* mutant compared to WT under salt and control conditions (**Fig. 10A**). These results validate our RNA-seq data that SCR106 involved in the transcriptional regulation of vast set of genes. This is further verified by the splicing patterns analysis of as set of randomly selected genes whose splicing is inhibited in the *scr106* mutant (**Fig. 10B**). We found an enrichment of introns in the *scr106* compared to the WT seedlings under salt and control conditions. Interestingly, for some genes like *OsUCIP3* (*Os04g0185500*), *OsCCZ1* (*Os08g0427300*), and *OsICS1* (*Os09g0361500*), the transcript levels of retained introns were even higher under control conditions in *scr106* mutant. This indicates that the *scr106* also regulates the pre-mRNA splicing during the development of rice seedling irrespective of any environmental stress (**Fig.**

10B). Overall, these results validate our RNA-seq data and confirm that SCR106 is an important splicing factor that regulate the splicing of a vast set of genes.

Discussion

Alternative splicing (AS) is an important gene regulatory process that generates multiple RNA isoforms from a single gene sequence. In plants, the SR proteins regulate the splice selection under certain developmental and stress conditions to generate specific RNA isoforms. Thus, AS regulated by SR proteins fine tunes the physiology and metabolism to cope with abiotic stress conditions like cold, heat, drought, high salinity, and heavy metals. SR proteins are characterized by their ability to interact simultaneously with RNA and other protein components via an RNA recognition motif (RRM) and through a domain rich in arginine and serine residues, the RS domain (Barta, Kalyna and Reddy, 2010; Shepard and Hertel, 2009) . In this study we identified *LOC_Os01g01150* in rice, which fulfils these criteria. We designated it as *OsSCR106*, because it groups with the SC subfamily of SR proteins and the protein has a size of 106 kDa. Base on the amino acid comparison using BLAST, OsSCR106 is a homolog of human SRSF11 and Arabidopsis IRR (At3g23900) (**Fig. S1**). To our surprise this *OsSCR106* was not considered before for RNA splicing studies in plants. It is important to note that we used BLAST to discover homologs of SRSF11 in Arabidopsis first, which revealed At3g23900, then by BLAST compared At3g23900 to rice proteome to get OsSCR106, which could have been missed if compared SRSF11 directly to the rice proteome. The SR proteins are prominent components of nuclear speckles, which occur throughout the nucleus, and presumably are the sites for pre-mRNA splicing. (Hackmann *et al.*, 2014; Shepard and Hertel, 2009). For OsSCR106, we observed a localization to nuclear speckles (**Fig. 1E**), suggesting a role for RNA splicing.

For further studies, we generated the k/o mutant *scr106*. In our analysis, the mutant seedlings showed reduced root and shoot lengths (**Fig. 3**). In contrast to animals, the loss-of-function mutants of SR proteins in plant are not embryonically lethal. The Arabidopsis *sr45-1* mutant showed many developmental abnormalities including delayed flowering, narrow leaves, and disturbed numbers of petals and stamens, and

reduced root growth (Ali *et al.*, 2007b). Moreover, even *sr* quadruple mutant (*sr34 sr34a sr34b sr30*), *rs* quadruple mutant (*rs31 rs31a rs40 rs41*), *rsz-rs2z* quintuple mutant (*rsz21 rsz22a rsz22 rs2z32 rs2z33*), and *sc35-scl* quintuple mutant (*scl28 scl30 scl30a scl33 sc35*) were not lethal in Arabidopsis (Yan *et al.*, 2017) . The loss of functions of SC35 and SCL (SC35-like, SCL28, SCL30, SCL30a and SCL33) results in pleiotropic changes in development, including serrate rosettes, late flowering, shorter roots, and anomalous phyllotaxis arrangement (Yan *et al.*, 2017). In addition, the overexpression of *AtRSZ33* caused changes in embryo and stomata development, cell expansion and shape, meristem formation, and decreased seed set (Kalyna, Lopato and Barta, 2003). Collectively, these examples confirm the role that SR proteins play in plant development.

Environmental stress regulation in plants is a complex phenomenon and many of the stress-related genes susceptible to alternative splicing (Chaudhary *et al.*, 2019; Filichkin *et al.*, 2015; Ganie and Reddy, 2021; Jabre *et al.*, 2019; Laloum, Martin and Duque, 2018; Ling, Mahfouz and Zhou, 2021; Rambout, Dequiedt and Maquat, 2018; Reddy *et al.*, 2013; Shang, Cao and Ma, 2017; Zhang *et al.*, 2019; Zheng *et al.*, 2019). The SR or SR-like proteins play crucial role during the regulation of AS thus orchestrate the plant stress responses (Albaqami, Laluk and Reddy, 2019; Butt *et al.*, 2021; Butt, 2022; Carvalho *et al.*, 2016; de Francisco Amorim *et al.*, 2018; Gu *et al.*, 2018; Laloum *et al.*, 2023; Liu *et al.*, 2016; Lu *et al.*, 2020; Muthusamy *et al.*, 2020; Park *et al.*, 2020; Yan *et al.*, 2017). Strikingly, the *scr106* mutant showed hypersensitive phenotypes to salt, ABA, mannitol (**Fig. 3**) and low-temperature treatments (**Fig. 4**). This is in line with the previous studies, where *sr45-1*, a loss-of-function, mutant observed defective response to glucose and ABA, and was hypersensitivity in response to salt stress (Albaqami, Laluk and Reddy, 2019; Carvalho, Carvalho and Duque, 2010). Butt et al. (2022) showed that the *rs33* loss-of-function rice mutant was hypersensitive to salt and low-temperature stresses, and that *RS33* regulates pre-mRNA splicing in response to abiotic stresses (Butt, 2022). Similarly, the *sr34b* mutant displayed a shorter root phenotype in response to cadmium treatment, with higher accumulation of cadmium in the root of *sr34b* in comparison to WT (Zhang *et al.*, 2014). Moreover, the loss of

function mutant *scl30a-1* was hypersensitive under ABA and salt stress during seed germination, whereas *SCL30a* overexpression reduced the sensitivity to ABA and enhanced salt stress tolerance (Laloum *et al.*, 2021). We analyzed the expression of OsSCR106 under different stress conditions and found that only cold treatment significantly affects the expression of OsSCR106 (**Fig. S4**). The SR proteins are showing different levels of expression and splicing patterns under abiotic stress conditions (Cruz *et al.*, 2014; Palusa, Ali and Reddy, 2007). Mostly the members of the plant-specific SR genes are affected by stress conditions while the expression of some of the SR genes is not affected. Thus the hypersensitivity response of *scr106* mutant against salt- and ABA-stress might be due to the activation/ repression of the stress-related genes which are regulated by the *OsSCR106*. For the detailed analysis, we retrieved the list of stress-related genes from the RAP database and compared with DEG and DAS genes. To our surprise, there are a large set of stress-related genes which are affected by the *OsSCR106* (**Table S1, Table S2**). Under control conditions the 94 stress-related genes up-regulated and 63 were downregulated while under salt stress 177 stress-related genes up-regulated and 296 were downregulated (**Table S1**). Similarly, splicing patterns were also changed for stress-related genes, 252 and 256 genes were differentially spliced under control and salt stress conditions respectively (**Table S2**). These data reinforce the crucial role of *OsSCR106* in plant stress regulation.

In plants, intron retention (RI) is the dominant AS event (Kumar *et al.*, 2022). Surprisingly, in our analysis of the *scr106* mutant, A3SS is a dominant event, both under control and salt stress conditions, which indicates that the OsSCR106 is mainly involved in regulating 3´ splice site selection. In a typical splicing reaction, spliceosome assembly begins with the base-pairing of the U1 snRNP with the 5′SS of the intron in the pre-mRNA strand. This binding is supported by the proteins of U1 snRNP and proteins of the serine-arginine-rich (SR) family (Wahl, Will and Luhrmann, 2009). This initial assembly also involves the interaction of U2 auxiliary factor (U2AF) to the branch point (BP) and the polypyrimidine tract just downstream of the BP. In eukaryotes, splicing factor 1 (SF1) interacts with the 65 kDa subunit of U2AF (U2AF65) through its

C-terminal RNA recognition motif (RRM). The smaller subunit of U2AF35 binds the AG dinucleotide of the 3′SS and play crucial roles in the recognition of 3′SS of an intron (Wahl, Will and Luhrmann, 2009; Wu *et al.*, 1999). In Arabidopsis, a mutation in the *AtSF1* induced pleiotropic developmental defects, including early flowering and showed oversensitivity to ABA treatment (Jang *et al.*, 2014). The high number of mis-spliced events for the 3´ splice site in *scr106* mutant indicates that the OsSCR106 possibly interacts with the U2AF35 and regulates the 3´ splice site selection in rice.

Altogether, we identified and disclosed the function of *OsSCR106* as a novel splicing factor. The *OsSCR106* localizes to the nuclear speckles and regulates the pre-mRNA splicing. The *OsSCR106* plays a pivotal role in abiotic stress tolerance in rice. The accurate control over the splicing machinery and its components helps to develop crops adaptable to changing climatic conditions.

Supplementary data

Fig. S1. Protein alignments of OsSCR106 with Arabidopsis and human homologs.
Fig. S2. Phylogenetic analysis of OsSCR106 with rice SR proteins.
Fig. S3. Protein alignments of OsSCR106 with rice SC-subfamily proteins.
Fig. S4. Gene expression of OsSCR106 under different stress conditions.
Table S1. Number of Stress-related DEG per comparison
Table S2. Number and type of stress-related AS events per comparison
Table S3. List of sequences used in this study
Dataset S1. List of DEG SR-C_vs_WT-C
Dataset S2. List of DEG SR-N_vs_SR-C
Dataset S3. List of DEG WT-N_vs_WT-C
Dataset S4. List of DAS SR-C_vs_WT-C
Dataset S5. List of DAS SR-N_vs_SR-C
Dataset S6. List of DAS WT-N_vs_WT-C
Dataset S7. List of stress-related DEG under control
Dataset S8. List of stress-related DEG under salt
Dataset S9. List of stress-related DAS under control
Dataset S10. List of stress-related DAS under salt

Acknowledgments

We would like to thank members of the genome engineering and synthetic biology laboratory at KAUST for their critical discussion and technical help in this work.

Author contributions

MM conceived the project. AA, HB, and GK designed and conducted the experiments. AA and HB analyzed the data. AA, HB, and MM designed and analyzed the splicing experiments. AA, HB, GK, IB, and MM wrote the paper. All authors read and approved the final manuscript.

Conflict of interest

'No conflict of interest declared'.

Funding

This work is funded by KAUST-baseline funding to Magdy Mahfouz.

Data availability

All data supporting the findings of this study are available within the paper and its supplementary materials published online. RNA-seq data that support the findings of this study have been deposited in NCBI Bioproject database.

The RAW data and processed data files were deposited in GEO (https://www.ncbi.nlm.nih.gov/geo/) under record **GSE232160**.

The following secure token has been created to allow review of record **GSE232160** while it remains in private status: **ehgrmyeevxwpfop**

Please note the following points:

- This token allows anonymous, read-only access to record **GSE232160** and associated accessions while they are private.
- Treat the token (**ehgrmyeevxwpfop**) as you would a password and realize that the token provides access to **GSE232160** to anyone who uses it.

To review GEO accession GSE232160:

Go to:

https://urldefense.com/v3/__https://www.ncbi.nlm.nih.gov/geo/query/acc.cgi?acc=GSE232160__;!!Nmw4Hv0!32DINo222hCumSHFxo50TFHdSBN2J2n7sQgN5Zf66c6iSfuWZdODgEkYdTOmOfQ24kVEtx5YLlXzh3SvuiCzavJcQLQ$

Enter token **ehgrmyeevxwpfop** into the box

References

Albaqami M, Laluk K, Reddy ASN. 2019. The Arabidopsis splicing regulator SR45 confers salt tolerance in a splice isoform-dependent manner. Plant Mol Biol **100**, 379-390.

Ali GS, Palusa SG, Golovkin M, Prasad J, Manley JL, Reddy AS. 2007a. Regulation of plant developmental processes by a novel splicing factor. PLoS One **2**, e471.

Ali GS, Palusa SG, Golovkin M, Prasad J, Manley JL, Reddy ASN. 2007b. Regulation of Plant Developmental Processes by a Novel Splicing Factor. PLoS One **2**.

AlShareef S, Ling Y, Butt H, Mariappan KG, Benhamed M, Mahfouz MM. 2017. Herboxidiene triggers splicing repression and abiotic stress responses in plants. BMC Genomics **18**, 260.

Barbosa-Morais NL, Carmo-Fonseca M, Aparicio S. 2006. Systematic genome-wide annotation of spliceosomal proteins reveals differential gene family expansion. Genome Res **16**, 66-77.

Barta A, Kalyna M, Reddy AS. 2010. Implementing a rational and consistent nomenclature for serine/arginine-rich protein splicing factors (SR proteins) in plants. Plant Cell **22**, 2926-2929.

Birney E, Kumar S, Krainer AR. 1993. Analysis of the RNA-recognition motif and RS and RGG domains: conservation in metazoan pre-mRNA splicing factors. Nucleic Acids Res **21**, 5803-5816.

Busch A, Hertel KJ. 2012. Evolution of SR protein and hnRNP splicing regulatory factors. Wiley Interdiscip Rev RNA **3**, 1-12.

Butt H, Bazin J, Alshareef S, Eid A, Benhamed M, Reddy ASN, Crespi M, Mahfouz MM. 2021. Overlapping roles of spliceosomal components SF3B1 and PHF5A in rice splicing regulation. Commun Biol **4**, 529.

Butt H, Eid A, Momin AA, Bazin J, Crespi M, Arold ST, Mahfouz MM. 2019a. CRISPR directed evolution of the spliceosome for resistance to splicing inhibitors. Genome Biol **20**, 73.

Butt H, Piatek A, Li L, A SNR, M MM. 2019b. Multiplex CRISPR Mutagenesis of the Serine/Arginine-Rich (SR) Gene Family in Rice. Genes (Basel) **10**.

Butt HB, J.; Prasad, K.V.S.K.; Awad, N.; Crespi, M.; Reddy, A.S.N.; Mahfouz, M.M. 2022. The Rice Serine/Arginine Splicing Factor RS33 Regulates Pre-mRNA Splicing during Abiotic Stress Responses. Cells **11, 1796**.

Caceres JF, Misteli T, Screaton GR, Spector DL, Krainer AR. 1997. Role of the modular domains of SR proteins in subnuclear localization and alternative splicing specificity. Journal of Cell Biology **138**, 225-238.

Caceres JF, Screaton GR, Krainer AR. 1998. A specific subset of SR proteins shuttles continuously between the nucleus and the cytoplasm. Genes & Development **12**, 55-66.

Calixto CPG, Guo W, James AB, Tzioutziou NA, Entizne JC, Panter PE, Knight H, Nimmo HG, Zhang R, Brown JWS. 2018. Rapid and Dynamic Alternative Splicing Impacts the Arabidopsis Cold Response Transcriptome. Plant Cell **30**, 1424-1444.

Carvalho RF, Carvalho SD, Duque P. 2010. The plant-specific SR45 protein negatively regulates glucose and ABA signaling during early seedling development in Arabidopsis. Plant Physiol **154**, 772-783.

Carvalho RF, Feijao CV, Duque P. 2013. On the physiological significance of alternative splicing events in higher plants. Protoplasma **250**, 639-650.

Carvalho RF, Szakonyi D, Simpson CG, Barbosa IC, Brown JW, Baena-Gonzalez E, Duque P. 2016. The Arabidopsis SR45 Splicing Factor, a Negative Regulator of Sugar Signaling, Modulates SNF1-Related Protein Kinase 1 Stability. Plant Cell **28**, 1910-1925.
Chaudhary S, Jabre I, Reddy ASN, Staiger D, Syed NH. 2019. Perspective on Alternative Splicing and Proteome Complexity in Plants. Trends Plant Sci **24**, 496-506.
Chen S, Li J, Liu Y, Li H. 2019. Genome-Wide Analysis of Serine/Arginine-Rich Protein Family in Wheat and Brachypodium distachyon. Plants (Basel) **8**.
Chen T, Cui P, Chen H, Ali S, Zhang S, Xiong L. 2013. A KH-domain RNA-binding protein interacts with FIERY2/CTD phosphatase-like 1 and splicing factors and is important for pre-mRNA splicing in Arabidopsis. PLoS Genet **9**, e1003875.
Cruz TM, Carvalho RF, Richardson DN, Duque P. 2014. Abscisic acid (ABA) regulation of Arabidopsis SR protein gene expression. Int J Mol Sci **15**, 17541-17564.
de Francisco Amorim M, Willing EM, Szabo EX, Francisco-Mangilet AG, Droste-Borel I, Macek B, Schneeberger K, Laubinger S. 2018. The U1 snRNP Subunit LUC7 Modulates Plant Development and Stress Responses via Regulation of Alternative Splicing. Plant Cell **30**, 2838-2854.
Di Zhang M-XC, Fu-Yuan Zhu, Jianhua Zhang & Ying-Gao Liu. 2020. Emerging Functions of Plant Serine/Arginine-Rich (SR) Proteins: Lessons from Animals. Critical Reviews in Plant Sciences **39, 2020**, 173-194
Dong C, He F, Berkowitz O, Liu J, Cao P, Tang M, Shi H, Wang W, Li Q, Shen Z, Whelan J, Zheng L. 2018. Alternative Splicing Plays a Critical Role in Maintaining Mineral Nutrient Homeostasis in Rice (Oryza sativa). Plant Cell **30**, 2267-2285.
Dressano K, Weckwerth PR, Poretsky E, Takahashi Y, Villarreal C, Shen Z, Schroeder JI, Briggs SP, Huffaker A. 2020. Dynamic regulation of Pep-induced immunity through post-translational control of defence transcript splicing. Nat Plants **6**, 1008-1019.
Duque P. 2011. A role for SR proteins in plant stress responses. Plant Signal Behav **6**, 49-54.
Filichkin S, Priest HD, Megraw M, Mockler TC. 2015. Alternative splicing in plants: directing traffic at the crossroads of adaptation and environmental stress. Current Opinion in Plant Biology **24**, 125-135.
Galganski L, Urbanek MO, Krzyzosiak WJ. 2017. Nuclear speckles: molecular organization, biological function and role in disease. Nucleic Acids Res **45**, 10350-10368.
Ganie SA, Reddy ASN. 2021. Stress-Induced Changes in Alternative Splicing Landscape in Rice: Functional Significance of Splice Isoforms in Stress Tolerance. Biology (Basel) **10**.
Gehring NH, Roignant JY. 2021. Anything but Ordinary - Emerging Splicing Mechanisms in Eukaryotic Gene Regulation. Trends Genet **37**, 355-372.
Golovkin M, Reddy AS. 1998. The plant U1 small nuclear ribonucleoprotein particle 70K protein interacts with two novel serine/arginine-rich proteins. Plant Cell **10**, 1637-1648.
Gu J, Xia Z, Luo Y, Jiang X, Qian B, Xie H, Zhu JK, Xiong L, Zhu J, Wang ZY. 2018. Spliceosomal protein U1A is involved in alternative splicing and salt stress tolerance in Arabidopsis thaliana. Nucleic Acids Res **46**, 1777-1792.
Hackmann A, Wu H, Schneider UM, Meyer K, Jung K, Krebber H. 2014. Quality control of spliced mRNAs requires the shuttling SR proteins Gbp2 and Hrb1. Nat Commun **5**, 3123.
Hiei Y, Komari T. 2008. Agrobacterium-mediated transformation of rice using immature embryos or calli induced from mature seed. Nat Protoc **3**, 824-834.

Isshiki M, Tsumoto A, Shimamoto K. 2006. The serine/arginine-rich protein family in rice plays important roles in constitutive and alternative splicing of pre-mRNA. Plant Cell **18**, 146-158.

Jabre I, Reddy ASN, Kalyna M, Chaudhary S, Khokhar W, Byrne LJ, Wilson CM, Syed NH. 2019. Does co-transcriptional regulation of alternative splicing mediate plant stress responses? Nucleic Acids Res **47**, 2716-2726.

Jang YH, Park HY, Lee KC, Thu MP, Kim SK, Suh MC, Kang H, Kim JK. 2014. A homolog of splicing factor SF1 is essential for development and is involved in the alternative splicing of pre-mRNA in Arabidopsis thaliana. Plant Journal **78**, 591-603.

Jeong S. 2017. SR Proteins: Binders, Regulators, and Connectors of RNA. Mol Cells **40**, 1-9.

Jiang JF, Liu XN, Liu CH, Liu GT, Li SH, Wang LJ. 2017. Integrating Omics and Alternative Splicing Reveals Insights into Grape Response to High Temperature. Plant Physiology **173**, 1502-1518.

Kalyna M, Lopato S, Barta A. 2003. Ectopic expression of atRSZ33 reveals its function in splicing and causes pleiotropic changes in development. Molecular Biology of the Cell **14**, 3565-3577.

Karimi M, Inze D, Depicker A. 2002. GATEWAY vectors for Agrobacterium-mediated plant transformation. Trends Plant Sci **7**, 193-195.

Keren H, Lev-Maor G, Ast G. 2010. Alternative splicing and evolution: diversification, exon definition and function. Nat Rev Genet **11**, 345-355.

Kumar K, Sinha SK, Maity U, Kirti PB, Kumar KRR. 2022. Insights into established and emerging roles of SR protein family in plants and animals. Wiley Interdisciplinary Reviews-Rna.

Kurihara D, Mizuta Y, Sato Y, Higashiyama T. 2015. ClearSee: a rapid optical clearing reagent for whole-plant fluorescence imaging. Development **142**, 4168-4179.

Laloum T, Carvalho SD, Martin G, Richardson DN, Cruz TMD, Carvalho RF, Stecca KL, Kinney AJ, Zeidler M, Barbosa ICR, Duque P. 2023. The SCL30a SR protein regulates ABA-dependent seed traits and germination under stress. Plant Cell and Environment **46**, 2112-2127.

Laloum T, Carvalho SD, Martín G, Richardson DN, Cruz TMD, Carvalho RF, Stecca KL, Kinney AJ, Zeidler M, Barbosa ICR, Duque P. 2021. The plant-specific SCL30a SR protein regulates ABA-dependent seed traits and salt stress tolerance during germination. bioRxiv, 2021.2010.2013.464208.

Laloum T, Martin G, Duque P. 2018. Alternative Splicing Control of Abiotic Stress Responses. Trends Plant Sci **23**, 140-150.

Lazar G, Schaal T, Maniatis T, Goodman HM. 1995. Identification of a plant serine-arginine-rich protein similar to the mammalian splicing factor SF2/ASF. Proc Natl Acad Sci U S A **92**, 7672-7676.

Ling Y, Alshareef S, Butt H, Lozano-Juste J, Li L, Galal AA, Moustafa A, Momin AA, Tashkandi M, Richardson DN, Fujii H, Arold S, Rodriguez PL, Duque P, Mahfouz MM. 2017. Pre-mRNA splicing repression triggers abiotic stress signaling in plants. Plant J **89**, 291-309.

Ling Y, Mahfouz MM, Zhou SX. 2021. Pre-mRNA alternative splicing as a modulator for heat stress response in plants. Trends in Plant Science **26**, 1153-1170.

Liu J, Chen X, Liang X, Zhou X, Yang F, Liu J, He SY, Guo Z. 2016. Alternative Splicing of Rice WRKY62 and WRKY76 Transcription Factor Genes in Pathogen Defense. Plant Physiol **171**, 1427-1442.

Lu CA, Huang CK, Huang WS, Huang TS, Liu HY, Chen YF. 2020. DEAD-Box RNA Helicase 42 Plays a Critical Role in Pre-mRNA Splicing under Cold Stress. Plant Physiol **182**, 255-271.

Manley JL, Krainer AR. 2010. A rational nomenclature for serine/arginine-rich protein splicing factors (SR proteins). Genes Dev **24**, 1073-1074.
Marasco LE, Kornblihtt AR. 2022. The physiology of alternative splicing. Nature Reviews Molecular Cell Biology.
Misteli T, Caceres JF, Spector DL. 1997. The dynamics of a pre-mRNA splicing factor in living cells. Nature **387**, 523-527.
Morton M, AlTamimi N, Butt H, Reddy ASN, Mahfouz M. 2019. Serine/Arginine-rich protein family of splicing regulators: New approaches to study splice isoform functions. Plant Sci **283**, 127-134.
Muthusamy M, Yoon EK, Kim JA, Jeong MJ, Lee SI. 2020. Brassica Rapa SR45a Regulates Drought Tolerance via the Alternative Splicing of Target Genes. Genes (Basel) **11**.
Palusa SG, Ali GS, Reddy AS. 2007. Alternative splicing of pre-mRNAs of Arabidopsis serine/arginine-rich proteins: regulation by hormones and stresses. Plant J **49**, 1091-1107.
Park HJ, You YN, Lee A, Jung H, Jo SH, Oh N, Kim HS, Lee HJ, Kim JK, Kim YS, Jung C, Cho HS. 2020. OsFKBP20-1b interacts with the splicing factor OsSR45 and participates in the environmental stress response at the post-transcriptional level in rice. Plant Journal **102**, 992-1007.
Plass M, Agirre E, Reyes D, Camara F, Eyras E. 2008. Co-evolution of the branch site and SR proteins in eukaryotes. Trends Genet **24**, 590-594.
Rambout X, Dequiedt F, Maquat LE. 2018. Beyond Transcription: Roles of Transcription Factors in Pre-mRNA Splicing. Chem Rev **118**, 4339-4364.
Rauch HB, Patrick TL, Klusman KM, Battistuzzi FU, Mei W, Brendel VP, Lal SK. 2014. Discovery and expression analysis of alternative splicing events conserved among plant SR proteins. Mol Biol Evol **31**, 605-613.
Reddy AS, Marquez Y, Kalyna M, Barta A. 2013. Complexity of the alternative splicing landscape in plants. Plant Cell **25**, 3657-3683.
Reddy AS, Shad Ali G. 2011. Plant serine/arginine-rich proteins: roles in precursor messenger RNA splicing, plant development, and stress responses. Wiley Interdiscip Rev RNA **2**, 875-889.
Richardson DN, Rogers MF, Labadorf A, Ben-Hur A, Guo H, Paterson AH, Reddy AS. 2011. Comparative analysis of serine/arginine-rich proteins across 27 eukaryotes: insights into sub-family classification and extent of alternative splicing. PLoS One **6**, e24542.
Shang XD, Cao Y, Ma LG. 2017. Alternative Splicing in Plant Genes: A Means of Regulating the Environmental Fitness of Plants. International Journal of Molecular Sciences **18**.
Shepard PJ, Hertel KJ. 2009. The SR protein family. Genome Biol **10**, 242.
Shi YG. 2017. Mechanistic insights into precursor messenger RNA splicing by the spliceosome. Nature Reviews Molecular Cell Biology **18**, 655-670.
Staiger D, Brown JWS. 2013. Alternative Splicing at the Intersection of Biological Timing, Development, and Stress Responses. Plant Cell **25**, 3640-3656.
Stamm S. 2008. Regulation of alternative splicing by reversible protein phosphorylation. J Biol Chem **283**, 1223-1227.
Wahl MC, Will CL, Luhrmann R. 2009. The Spliceosome: Design Principles of a Dynamic RNP Machine. Cell **136**, 701-718.
Will CL, Luhrmann R. 2011. Spliceosome structure and function. Cold Spring Harb Perspect Biol **3**.

Wu SP, Romfo CM, Nilsen TW, Green MR. 1999. Functional recognition of the 3 ' splice site AG by the splicing factor U2AF(35). Nature **402**, 832-835.
Wu X, Xu J, Meng X, Fang X, Xia M, Zhang J, Cao S, Fan T. 2022. Linker histone variant HIS1-3 and WRKY1 Oppositely Regulate Salt Stress Tolerance in Arabidopsis. Plant Physiology.
Xiang S, Gapsys V, Kim HY, Bessonov S, Hsiao HH, Mohlmann S, Klaukien V, Ficner R, Becker S, Urlaub H, Luhrmann R, de Groot B, Zweckstetter M. 2013. Phosphorylation drives a dynamic switch in serine/arginine-rich proteins. Structure **21**, 2162-2174.
Xie K, Minkenberg B, Yang Y. 2015. Boosting CRISPR/Cas9 multiplex editing capability with the endogenous tRNA-processing system. Proc Natl Acad Sci U S A **112**, 3570-3575.
Yan Q, Xia X, Sun Z, Fang Y. 2017. Depletion of Arabidopsis SC35 and SC35-like serine/arginine-rich proteins affects the transcription and splicing of a subset of genes. PLoS Genet **13**, e1006663.
Zhang P, Deng H, Mao FM, Liu YS. 2013. Alterations of Alternative Splicing Patterns of Ser/Arg-Rich (SR) Genes in Response to Hormones and Stresses Treatments in Different Ecotypes of Rice (Oryza sativa). Journal of Integrative Agriculture **12**, 737-748.
Zhang W, Du B, Liu D, Qi X. 2014. Splicing factor SR34b mutation reduces cadmium tolerance in Arabidopsis by regulating iron-regulated transporter 1 gene. Biochem Biophys Res Commun **455**, 312-317.
Zhang X, Yuan J, Zhang X, Liu C, Xiang J, Li F. 2019. Genome-Wide Analysis of Alternative Splicing Provides Insights Into Stress Response of the Pacific White Shrimp Litopenaeus vanname. Front Genet **10**, 845.
Zhang XN, Mount SM. 2009. Two alternatively spliced isoforms of the Arabidopsis SR45 protein have distinct roles during normal plant development. Plant Physiol **150**, 1450-1458.
Zheng J, Liu F, Zhu C, Li X, Dai X, Yang B, Zou X, Ma Y. 2019. Identification, expression, alternative splicing and functional analysis of pepper WRKY gene family in response to biotic and abiotic stresses. PLoS One **14**, e0219775.
Zhou Z, Fu XD. 2013. Regulation of splicing by SR proteins and SR protein-specific kinases. Chromosoma **122**, 191-207.

Figure Legends:

Fig. 1. Expression analysis and subcellular localization of OsSCR106 in rice.

(A) Domain organization of the OsSCR106 (SC-Related, LOC_Os01g01150) protein, generated using publically available online tool Prosite (https://prosite.expasy.org/). **(B)** Schematic diagram of the pKGWSF7 construct for expressing the *pOsSCR106::GUS*. The expression is driven by the endogenous promoter of *OsSCR106*. **(C)** GUS staining analysis of transgenic plants carrying *pOsSCR106::GUS*. **(D)** Schematic diagram of the pRGEB32 construct for expressing the *pUBI::OsSCR106:EGFP*. The expression is driven by the *OsUbiquitin* promoter (*pUBI*). **(E)** *pUBI::OsSCR106:EGFP* expression in the transition zone and the root tip of roots of xxx day old seedlings. **(F)** Magnification of *OsSCR106:EGFP* expressed under pUBI in rice roots; red arrows indicate nuclear speckles. Bar, 50 µM or 20 µM in white.

Fig. 2. Targeted mutagenesis of the rice *SCR106* locus.

(A) Schematic of *OsSCR106* gene structure and the target site. *OsSCR106* contains three exons. The translation initiation codon (ATG) (arrow) and termination codon (TGA) (asterisk) are shown. The target site nucleotides are shown in capital letters, and the protospacer adjacent motif (PAM) site is underlined. **(B)** Nucleotide sequences and translation at the target site 1 and 2 in the T_0 mutant rice plants. The recovered mutated allele sequences are shown. The PAM site is underlined. The red capital letter indicates the inserted nucleotides, and/or changed amino acid. "-/+" indicate the deletion or insertion of the indicated number of nucleotides. The chromatogram represent data obtained by Sanger sequencing.

Fig. 3. The *scr106* mutant is hypersensitive to salt, ABA, and mannitol.

(A, D, G, J) Root and shoot phenotype of WT and *scr106* mutant grown on ½ MS media for 7-days, under control conditions **(A)**, salt treatment **(D)**, ABA treatment **(G)**, or

mannitol treatment **(J)**. **(B, C, E, F, H, I, K, L)** Root and shoot length of WT and *scr106* mutant under control and stress treatment; organ length was calculated relative to root or shoot length of the WT plants grown on ½ MS media. The Student's t-test analysis indicated a significant difference compared with WT (*P<0.05, **P<0.01). Values are means ± SD of at least six biological replicates (represented as dots).

Fig. 4. The mutation of *SCR106* leads to hypersensitive phenotypes to low-temperature treatments.

(A-C). The seeds of WT and *scr106* mutant were germinated on ½MS media and grown at different low-temperatures. Seedlings were grown at (day/night) 28/26 ºC, 22/20 ºC, or 18/15 ºC for 12-days. At 16/14 ºC, or 4 ºC, seeds were initially treated at these temperatures for 14-days and then recovered for 5-days. The germination and growth of the *scr106* mutant is significantly delayed as compared to WT. The relative growth length was calculated based on a root or shoot length of 100% for plants of each genotype grown on ½ MS media at indicated temperatures. For 28/26 ºC, the control conditions, the relative growth length is calculated based on a root or shoot length of 100% for WT plants. The Student's t-test analysis indicated a significant difference compared with WT (*P<0.05, **P<0.01). Values are means ± SD of at least six biological replicates (represented as dots).

Fig. 5. The hypersensitive phenotype of *scr106* under NaCl and ABA treatment is restored by the expression of *OsSCR106*.

(A-C) WT, *scr106* mutant complemented with *pOsSCR106::OsSCR106-EGFP* (Comp) and *scr106* mutant grown for 7 days under control or stress conditions. Shoot and root length of WT, complemented *scr106* mutant and *scr106* mutant under control or stress conditions; length was calculated relative to root or shoot length of the WT plants grown on ½ MS media . The Student's t-test analysis indicated a significant difference compared with WT (*P<0.05, **P<0.01). Values are means ± SD of at least six biological replicates (represented as dots).

Fig. 6. The hypersensitive phenotype of *scr106* under low temperature is rescued by the expression of *OsSCR106*.

(A-C) The transformation of the *OsSCR106* cDNA rescued the hypersensitive phenotype under low temperature treatment. The relative growth length is calculated based on a root or shoot length of 100% for WT plants. The Student's t-test analysis indicated a significant difference compared with WT (*P<0.05, **P<0.01). Values are means ± SD of at least six biological replicates (represented as dots).

Fig. 7. Loss of *SCR106* affects the global gene expression and alternative gene splicing in rice.

(A) The number of up- and downregulated differentially expressed genes (DEGs) in *scr106* compared to WT. **(B)** Volcano plot represents the DEGs. Black dots represent the genes that are not significantly differentially expressed, while red and green dots are the genes that are significantly up- and down-regulated respectively. The gene IDs for top 10 highly differentially expressed are shown. **(C)** GO enrichment analysis of the DEGs regulated by *OsSCR106*. **(D)** Bar plot showing the differentially alternative splicing events (DAS) between the *scr106* and WT. The numbers of each type of event are given. The majority of these events are alternative 3´ splice site (A3SS), and intron retention (IR). SE, exon skipping; A5SS, alternative 5´ splice site; MXE, mutually exclusive exons. **(E)** GO enrichment analysis of the DAS genes regulated by *OsSCR106*. **(F)** Overlap of DEG with DAS events for *OsSCR106* and WT.

Fig. 8. Genome-wide transcriptional effects of *scr106* mutant under salt stress

(A) Number of differentially expressed genes (DEGs, up, and downregulated) in the *scr106* and WT under salt stress conditions. **(B)** An overlap between WT and *scr106*. A large number of the DEGs in WT show overlap with *scr106.* Majority of the DEGs are uniquely regulated by *OsSCR106* under salt stress conditions. **(C)** Volcano plot of the DEGs comparing *scr106* and WT under salt stress. Red dots represents upregulated genes while green dots denotes downregulated genes. Black dots represent the genes that are not significantly differentially expressed. The gene IDs for top 10 highly

differentially expressed are shown. **(D)** GO enrichment analysis of the DEGs regulated by *scr106* under salt stress conditions.

Fig. 9. *OsSCR106* alters the pre-mRNA splicing under salt stress

(A) Bar plot showing the number of each type of differential alternative splicing (DAS) event induced by salt treatment in the *scr106* mutant and the WT. **(B)** Venn diagram showing the overlap of differential alternative splicing (DAS) genes between *scr106* and WT plants under salt stress conditions. **(C)** GO enrichment analysis of the DAS regulated by *scr106* under salt stress.

Figure 10: Evaluation of differential expression and differential intron retention (IR) in *scr106* and wild-type (WT) plants.

Rice seedlings of the mutant *scr106* and WT were germinate under control and 125 mM NaCl for one-week. Total RNA extracted from the whole seedling was used for mRNA expression and splicing pattern analysis. **(A)** Validation of expression of genes detected by RNA-seq. Genes were randomly selected from the lists of DEG under control- or salt-treatment. The locus names are *OsRLCK318* (*Os11g0213000*), *OsHSP24.1* (*Os02g0758000*), *OsS40-1* (*Os05g0531100*), *OsNF-YA6* (*Os07g0608200*), *OsCYP71E5* (*Os12g0512800*), *OsSIET1* (*Os03g0107300*), and *OsIMA2* (*Os07g0142100*). Bars represent the mean ± SEM of three replicates. *OsActin* was used as an internal control. (Student's t-test; *$P < 0.05$, **$P < 0.01$). **(B)** Semiquantitative RT-PCR analysis to validate the IR of randomly selected genes from the lists of DAS under control- or salt-treatment. Arrowheads indicate splicing variants that changed in *scr106* mutant. The gene structures and retained introns are shown. Red boxes indicate the PCR fragments.

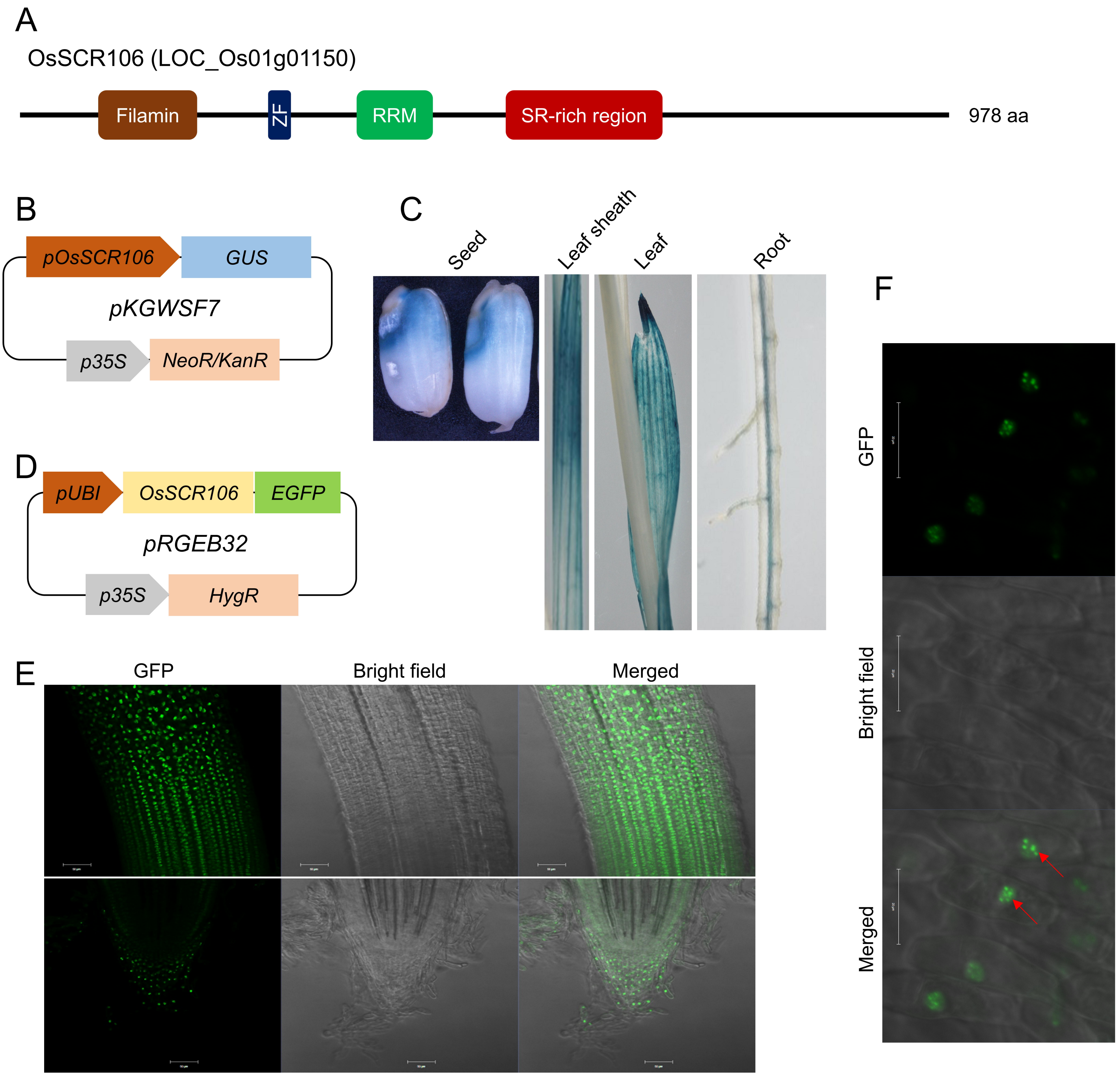

Fig. 1. Expression analysis and subcellular localization of OsSCR106 in rice.

(A) Domain organization of the OsSCR106 (SC-Related, LOC_Os01g01150) protein, generated using publically available online tool Prosite (https://prosite.expasy.org/). **(B)** Schematic diagram of the pKGWSF7 construct for expressing the *pOsSCR106::GUS*. The expression is driven by the endogenous promoter of *OsSCR106*. **(C)** GUS staining analysis of transgenic plants carrying *pOsSCR106::GUS*. **(D)** Schematic diagram of the pRGEB32 construct for expressing the *pUBI::OsSCR106:EGFP*. The expression is driven by the *OsUbiquitin* promoter (*pUBI*). **(E)** *pUBI::OsSCR106:EGFP* expression in the transition zone and the root tip of roots of xxx day old seedlings. **(F)** Magnification of *OsSCR106:EGFP* expressed under pUBI in rice roots; red arrows indicate nuclear speckles. Bar, 50 μM or 20 μM in white.

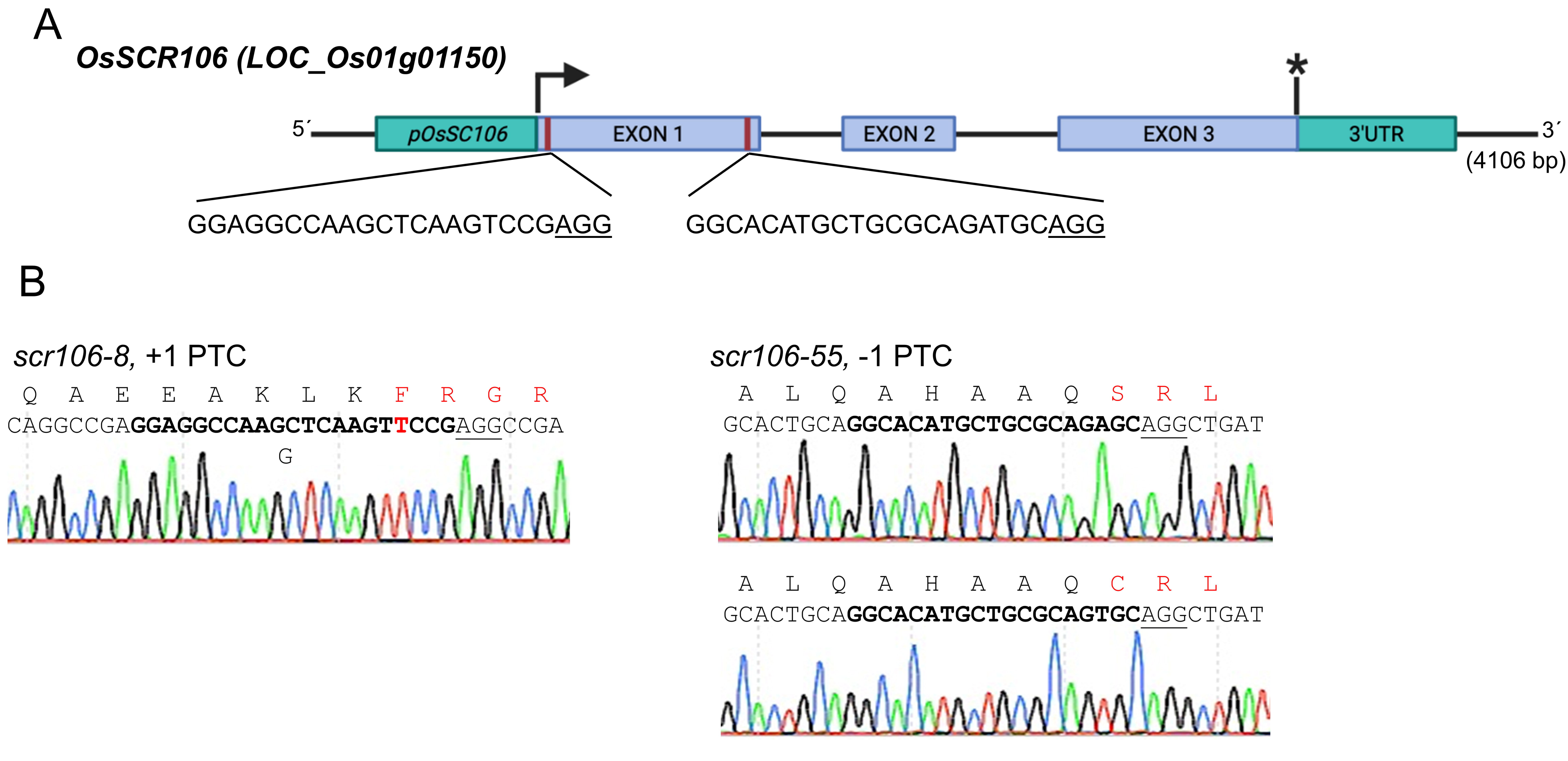

Fig. 2. Targeted mutagenesis of the rice *SCR106* locus.

(A) Schematic of *OsSCR106* gene structure and the target site. *OsSCR106* contains three exons. The translation initiation codon (ATG) (arrow) and termination codon (TGA) (asterisk) are shown. The target site nucleotides are shown in capital letters, and the protospacer adjacent motif (PAM) site is underlined. **(B)** Nucleotide sequences and translation at the target site 1 and 2 in the T_0 mutant rice plants. The recovered mutated allele sequences are shown. The PAM site is underlined. The red capital letter indicates the inserted nucleotides, and/or changed amino acid. “-/+” indicate the deletion or insertion of the indicated number of nucleotides. The chromatogram represent data obtained by Sanger sequencing.

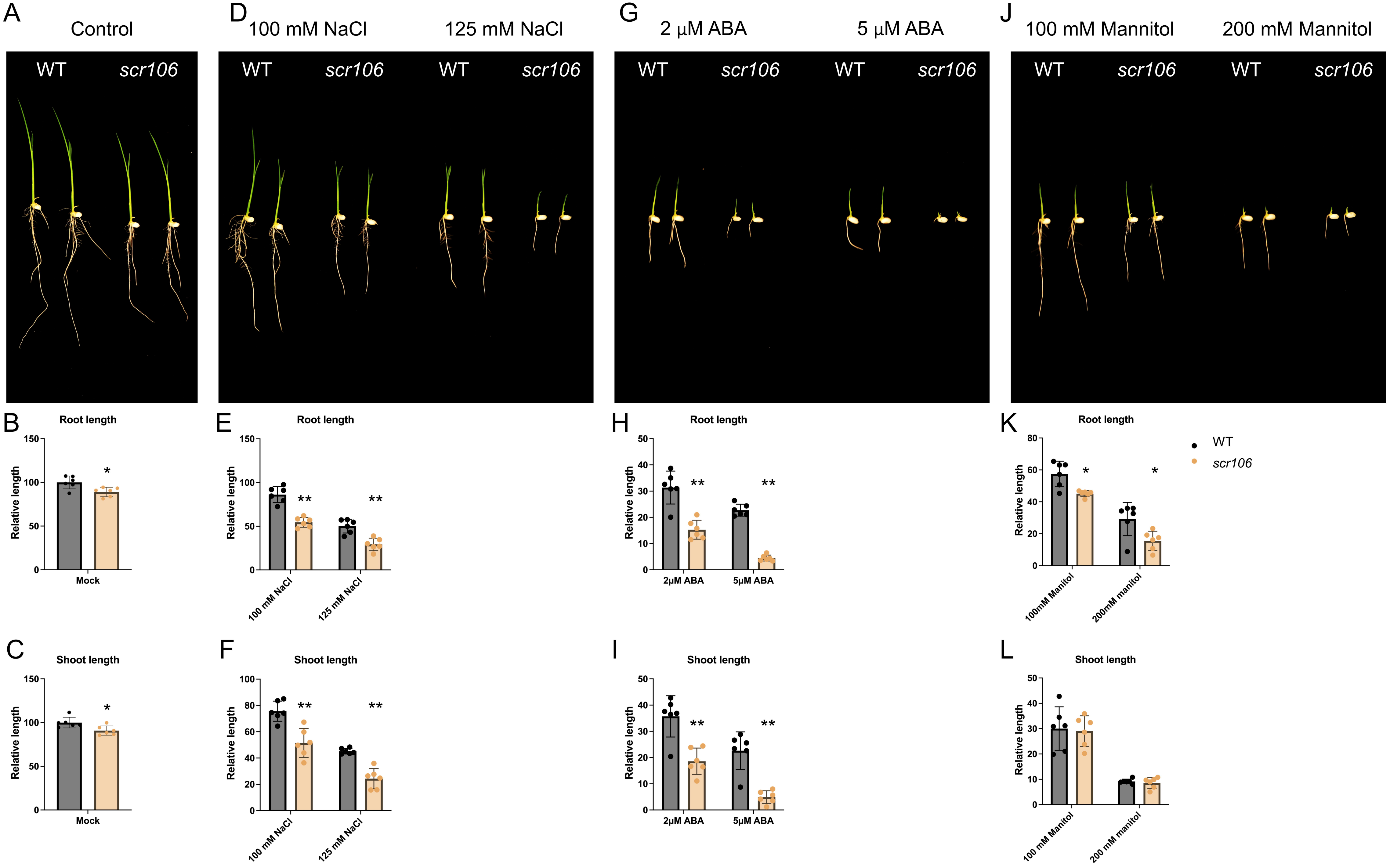

Fig. 3. The *scr106* mutant is hypersensitive to salt, ABA, and mannitol.

(A, D, G, J) Root and shoot phenotype of WT and *scr106* mutant grown on ½ MS media for 7-days, under control conditions **(A)**, salt treatment **(D)**, ABA treatment **(G)**, or mannitol treatment **(J)**. **(B, C, E, F, H, I, K, L)** Root and shoot length of WT and *scr106* mutant under control and stress treatment; organ length was calculated relative to root or shoot length of the WT plants grown on ½ MS media. The Student's t-test analysis indicated a significant difference compared with WT (*P<0.05, **P<0.01). Values are means ± SD of at least six biological replicates (represented as dots).

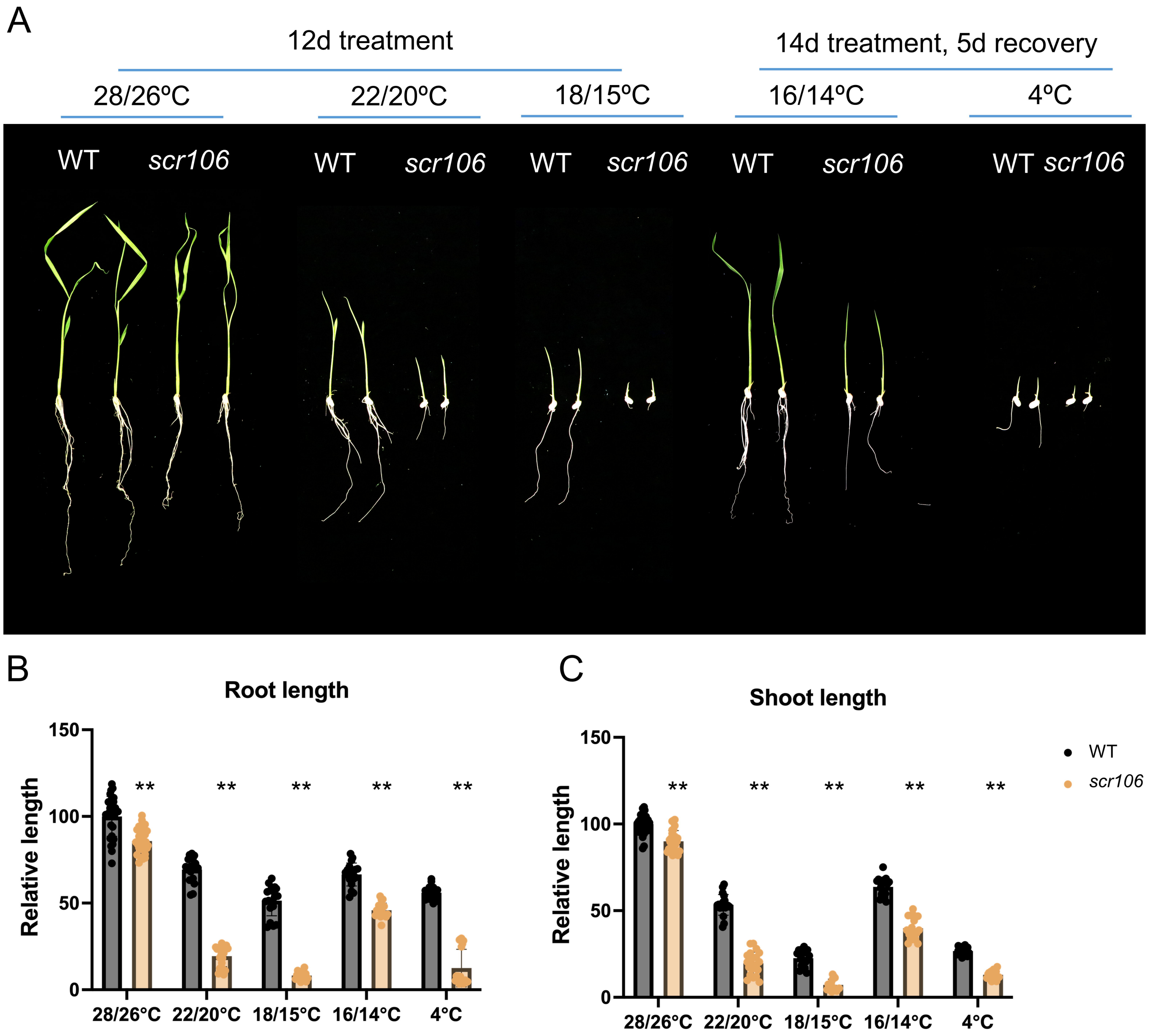

Fig. 4. The mutation of *SCR106* leads to hypersensitive phenotypes to low-temperature treatments.

(A-C). The seeds of WT and *scr106* mutant were germinated on ½MS media and grown at different low-temperatures. Seedlings were grown at (day/night) 28/26 ºC, 22/20 ºC, or 18/15 ºC for 12-days. At 16/14 ºC, or 4 ºC, seeds were initially treated at these temperatures for 14-days and then recovered for 5-days. The germination and growth of the *scr106* mutant is significantly delayed as compared to WT. The relative growth length was calculated based on a root or shoot length of 100% for plants of each genotype grown on ½ MS media at indicated temperatures. For 28/26 ºC, the control conditions, the relative growth length is calculated based on a root or shoot length of 100% for WT plants. The Student's t-test analysis indicated a significant difference compared with WT (*P<0.05, **P<0.01). Values are means ± SD of at least six biological replicates (represented as dots).

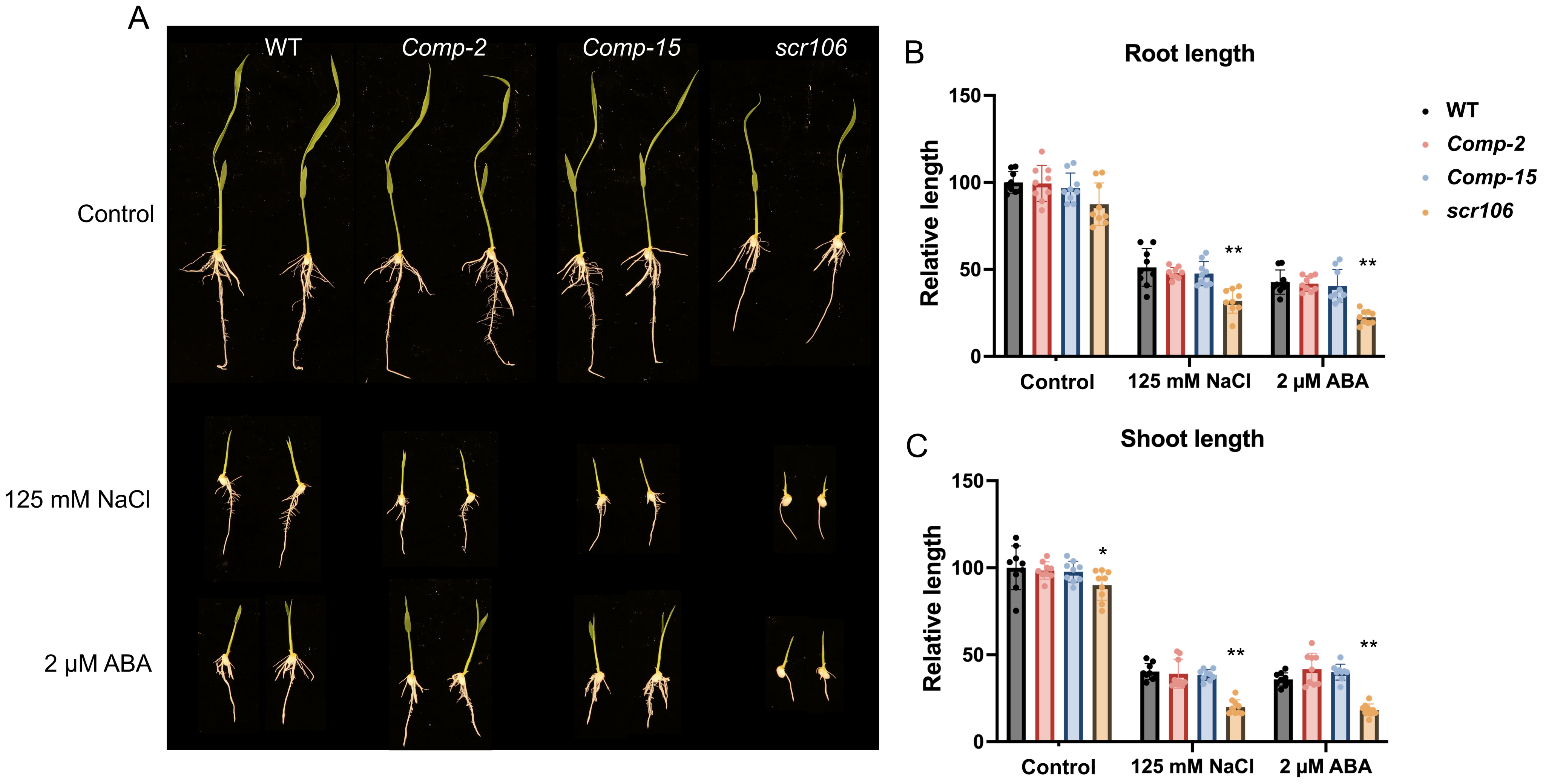

Fig. 5. The hypersensitive phenotype of *scr106* under NaCl and ABA treatment is restored by the expression of *OsSCR106*.

(A-C) WT, *scr106* mutant complemented with *pOsSCR106::OsSCR106-EGFP* (Comp) and *scr106* mutant grown for 7 days under control or stress conditions. Shoot and root length of WT, complemented *scr106* mutant and *scr106* mutant under control or stress conditions; length was calculated relative to root or shoot length of the WT plants grown on ½ MS media . The Student's t-test analysis indicated a significant difference compared with WT (*P<0.05, **P<0.01). Values are means ± SD of at least six biological replicates (represented as dots).

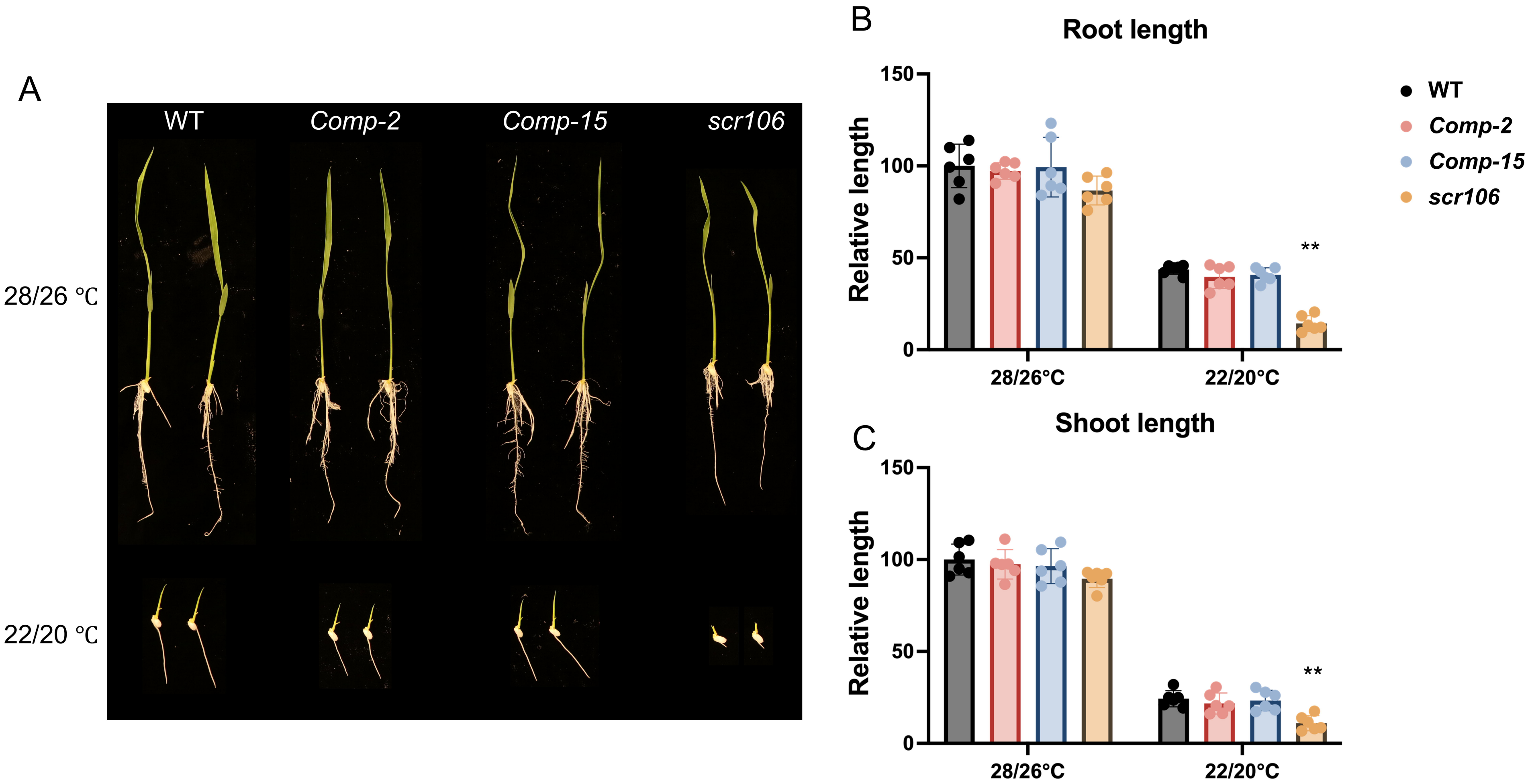

Fig. 6. The hypersensitive phenotype of *scr106* under low temperature is rescued by the expression of *OsSCR106*.

(A-C) The transformation of the *OsSCR106* cDNA rescued the hypersensitive phenotype under low temperature treatment. The relative growth length is calculated based on a root or shoot length of 100% for WT plants. The Student's t-test analysis indicated a significant difference compared with WT (*P<0.05, **P<0.01). Values are means ± SD of at least six biological replicates (represented as dots).

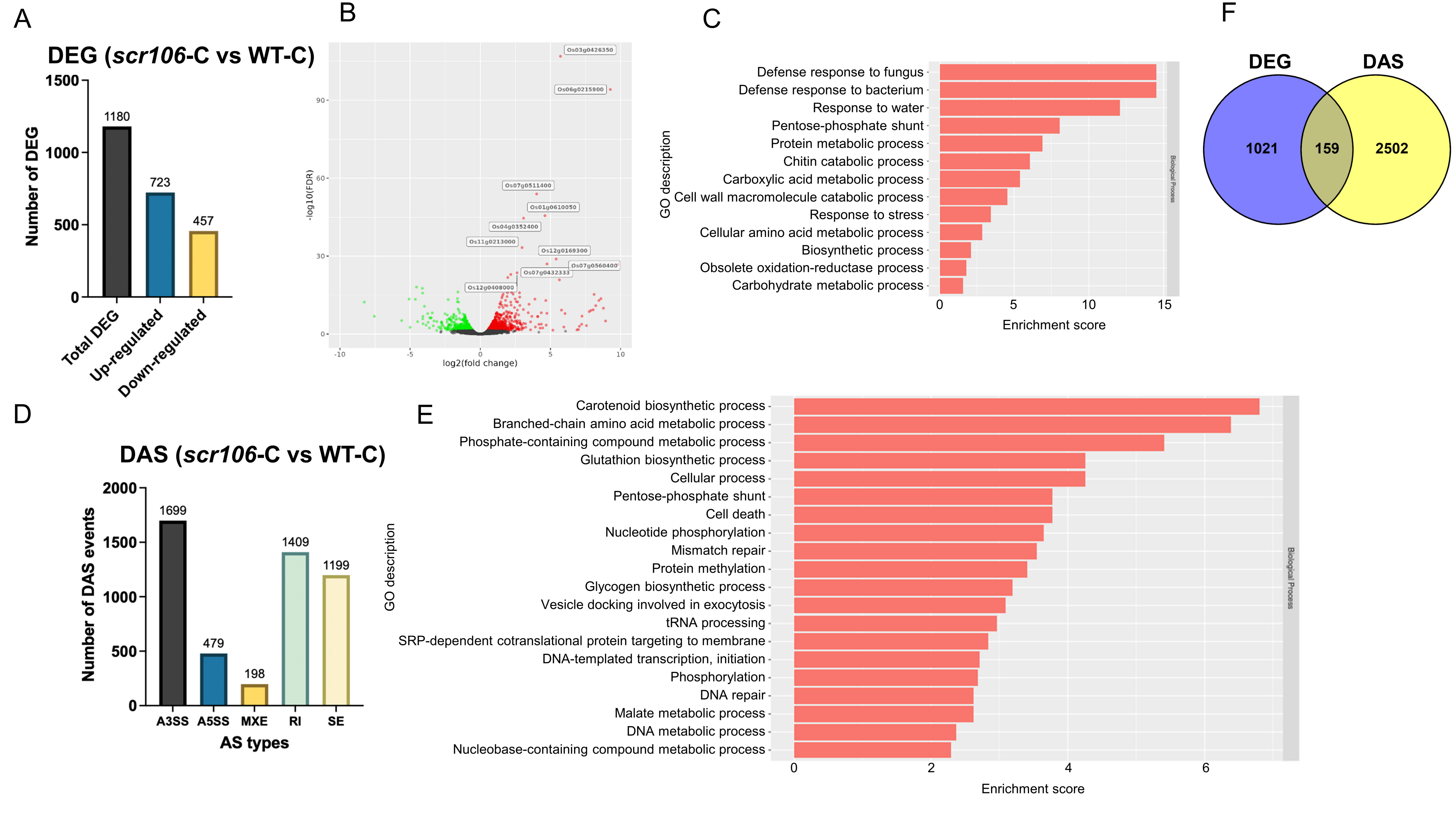

Fig. 7. Loss of *SCR106* affects the global gene expression and alternative gene splicing in rice.
(A) The number of up- and downregulated differentially expressed genes (DEGs) in *scr106* compared to WT. **(B)** Volcano plot represents the DEGs. Black dots represent the genes that are not significantly differentially expressed, while red and green dots are the genes that are significantly up- and down-regulated respectively. The gene IDs for top 10 highly differentially expressed are shown. **(C)** GO enrichment analysis of the DEGs regulated by *OsSCR106*. **(D)** Bar plot showing the differentially alternative splicing events (DAS) between the *scr106* and WT. The numbers of each type of event are given. The majority of these events are alternative 3′ splice site (A3SS), and intron retention (IR). SE, exon skipping; A5SS, alternative 5′ splice site; MXE, mutually exclusive exons. **(E)** GO enrichment analysis of the DAS genes regulated by *OsSCR106*. **(F)** Overlap of DEG with DAS events for *OsSCR106* and WT.

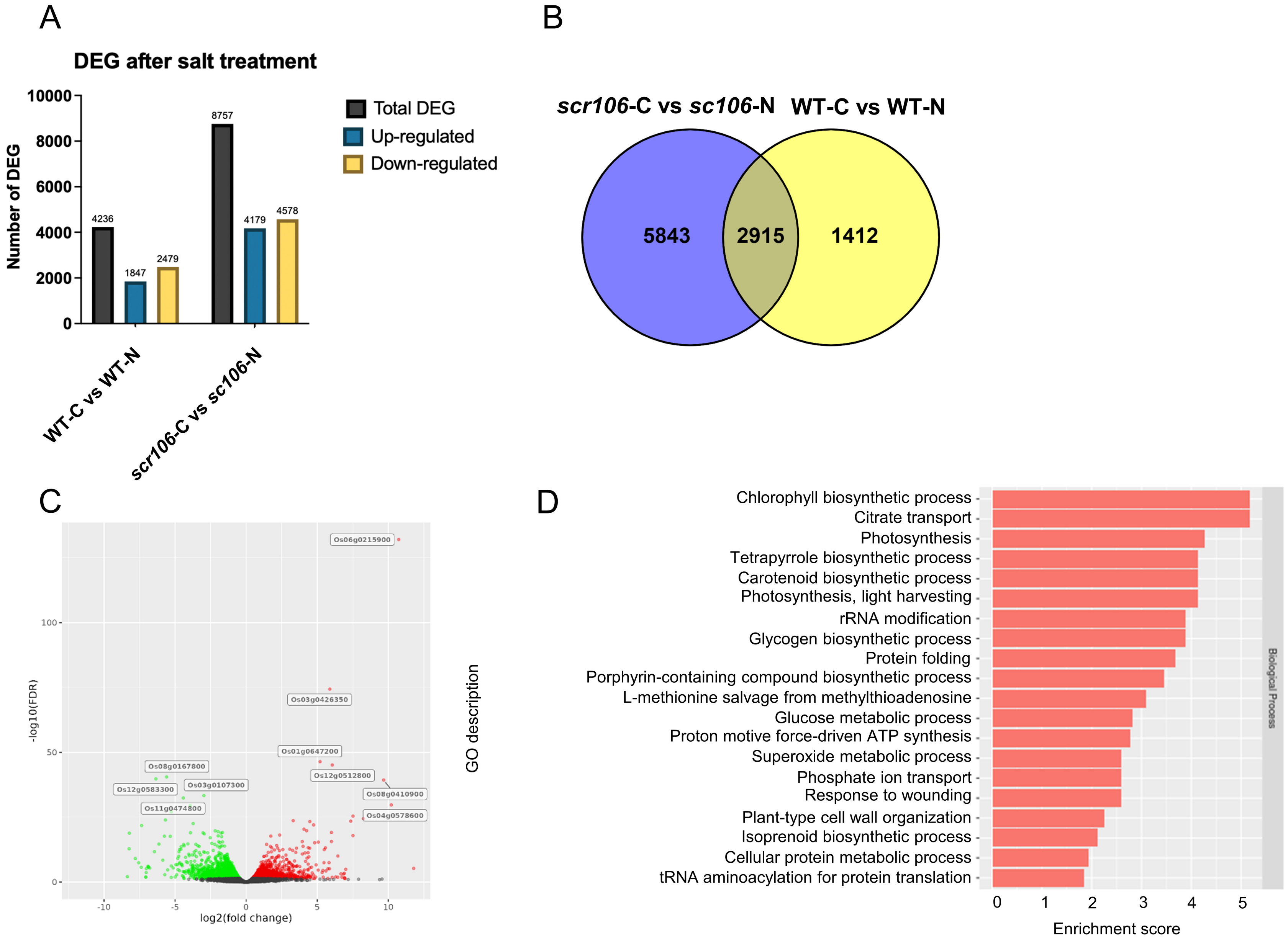

Fig. 8. Genome-wide transcriptional effects of *scr106* mutant under salt stress

(A) Number of differentially expressed genes (DEGs, up, and downregulated) in the *scr106* and WT under salt stress conditions. **(B)** An overlap between WT and *scr106*. A large number of the DEGs in WT show overlap with *scr106.* Majority of the DEGs are uniquely regulated by *OsSCR106* under salt stress conditions. **(C)** Volcano plot of the DEGs comparing *scr106* and WT under salt stress. Red dots represents upregulated genes while green dots denotes downregulated genes. Black dots represent the genes that are not significantly differentially expressed. The gene IDs for top 10 highly differentially expressed are shown. **(D)** GO enrichment analysis of the DEGs regulated by *scr106* under salt stress conditions.

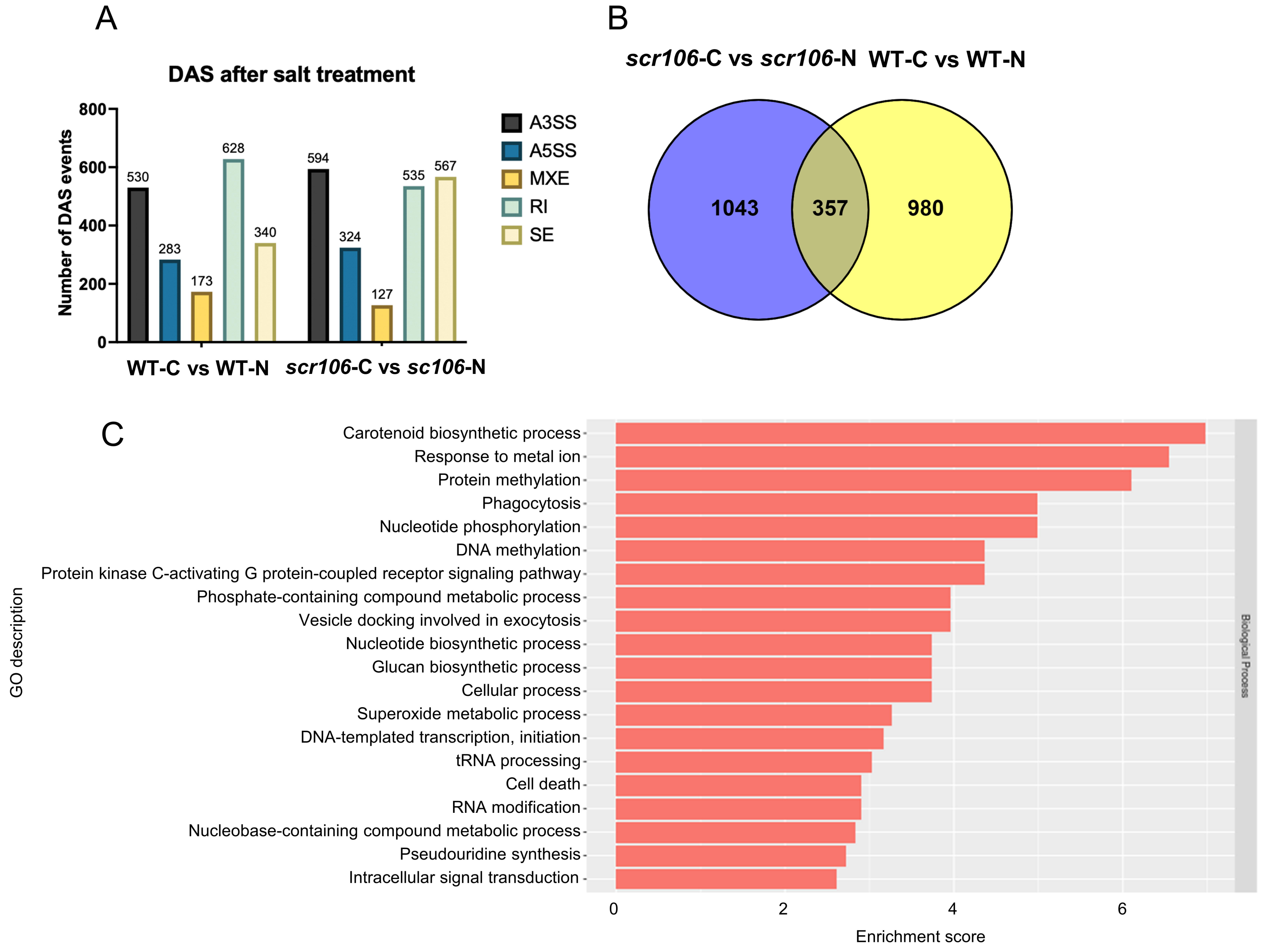

Fig. 9. *OsSCR106* alters the pre-mRNA splicing under salt stress

(A) Bar plot showing the number of each type of differential alternative splicing (DAS) event induced by salt treatment in the *scr106* mutant and the WT. **(B)** Venn diagram showing the overlap of differential alternative splicing (DAS) genes between *scr106* and WT plants under salt stress conditions. **(C)** GO enrichment analysis of the DAS regulated by *scr106* under salt stress.

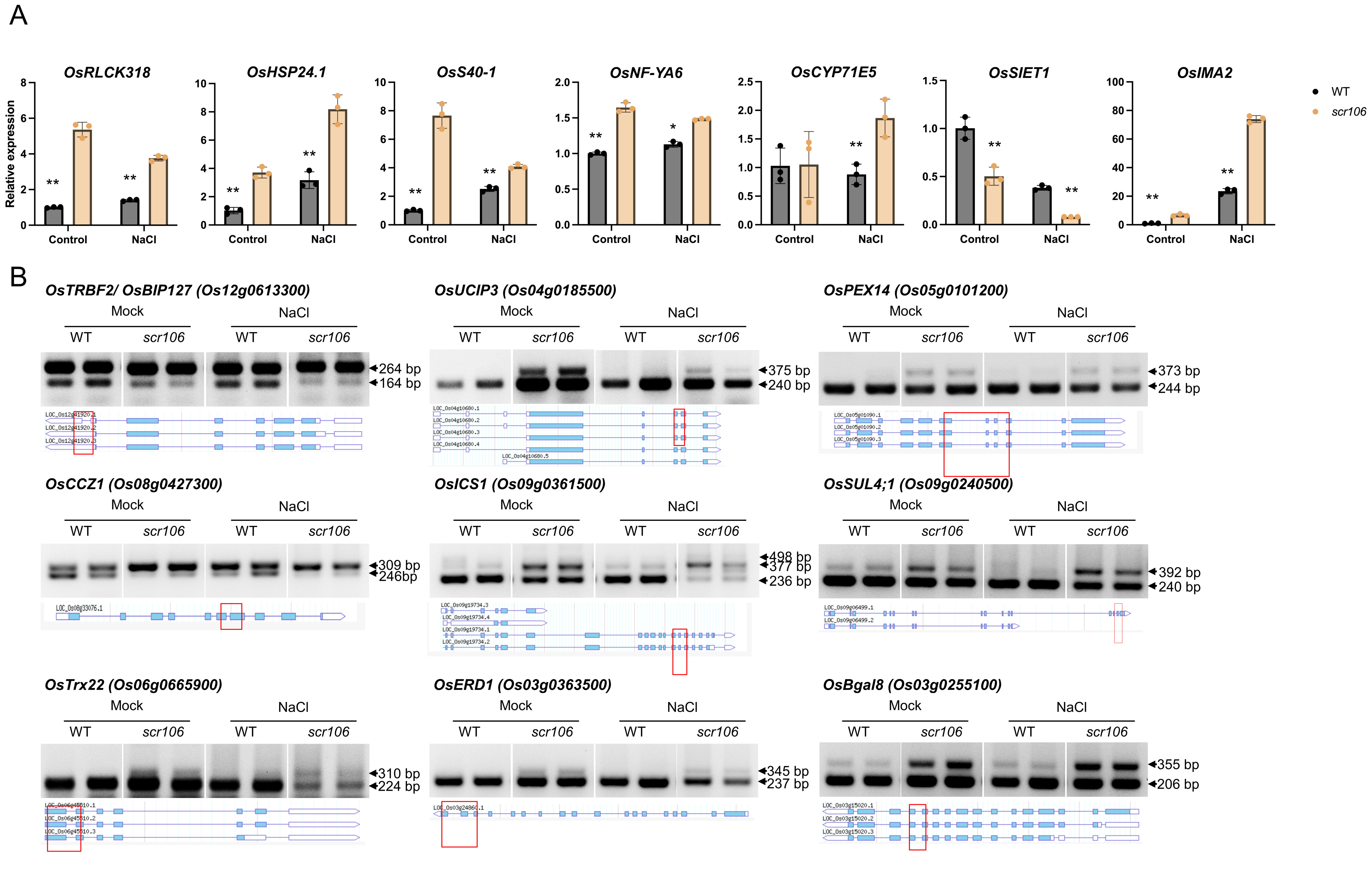

Figure 10: Evaluation of differential expression and differential intron retention (IR) in *scr106* and wild-type (WT) plants. Rice seedlings of the mutant *scr106* and WT were germinate under control and 125 mM NaCl for one-week. Total RNA extracted from the whole seedling was used for mRNA expression and splicing pattern analysis. **(A)** Validation of expression of genes detected by RNA-seq. Genes were randomly selected from the lists of DEG under control- or salt-treatment. The locus names are *OsRLCK318* (*Os11g0213000*), *OsHSP24.1* (*Os02g0758000*), *OsS40-1* (*Os05g0531100*), *OsNF-YA6* (*Os07g0608200*), *OsCYP71E5* (*Os12g0512800*), *OsSIET1* (*Os03g0107300*), and *OsIMA2* (*Os07g0142100*). Bars represent the mean ± SEM of three replicates. *OsActin* was used as an internal control. (Student's t-test; *P < 0.05, **P < 0.01). **(B)** Semiquantitative RT-PCR analysis to validate the IR of randomly selected genes from the lists of DAS under control- or salt-treatment. Arrowheads indicate splicing variants that changed in *scr106* mutant. The gene structures and retained introns are shown. Red boxes indicate the PCR fragments.
